# Decoding MicroRNA-Guided Antiviral Defense in Cucurbitaceae: Regulatory Networks, RNA Silencing Cross-Talk, and Emerging Strategies for Crop Resilience

**DOI:** 10.3390/ijms27146300

**Published:** 2026-07-15

**Authors:** Maksymilian Pisz, Agata Głuchowska, Zhimin Yin, Magdalena Pawełkowicz

**Affiliations:** 1 Department of Plant Genetics, Breeding and Biotechnology, Institute of Biology, Warsaw University of Life Sciences—SGGW, 159 Nowoursynowska Str., 02-776 Warsaw, Poland; maksymilian_pisz@sggw.edu.pl (M.P.); agata_gluchowska@sggw.edu.pl (A.G.); 2 Plant Breeding and Acclimatization Institute—National Research Institute in Radzików, Młochów Division, Department of Potato Genetics and Parental Lines, 19 Platanowa Str., 05-831 Młochów, Poland; z.yin@ihar.edu.pl

**Keywords:** microRNA, Cucurbitaceae, RNA-based crop protection, antiviral immunity, small RNAs, regulatory cross-talk, plant virology, crop resilience

## Abstract

MicroRNAs (miRNAs) are central regulators of gene expression and play pivotal roles in plant antiviral defense. In Cucurbitaceae, a globally important crop family including cucumber, melon, and watermelon, viral pathogens such as CGMMV, CMV, and ZYMV represent major constraints on productivity. However, the regulatory complexity of miRNA-mediated antiviral responses in these species remains incompletely understood. This review provides an integrated overview of recent advances in miRNA-guided antiviral immunity in Cucurbitaceae, highlighting the dynamic reprogramming of small RNA pathways upon viral infection. Conserved miRNA families act as key regulatory hubs, controlling development, hormone signaling, and defense responses, while viral suppressors interfere with RNA silencing machinery, reshaping host regulatory networks. Emerging evidence further reveals multilayered interactions between miRNAs and other non-coding RNAs, including lncRNAs and circRNAs, indicating complex cross-talk that fine-tunes antiviral responses in a species- and virus-specific manner. Importantly, miRNAs exhibit a dual role by contributing both to antiviral defense and to symptom development. Advances in artificial miRNAs and RNA-based technologies underscore their potential for engineering durable virus resistance. Overall, miRNA-centered regulatory networks represent a promising target for next-generation crop protection strategies in Cucurbitaceae.

## 1. Introduction

Cucurbitaceae represents one of the most economically important plant families worldwide, comprising major horticultural crops such as cucumber (*Cucumis sativus* L.), melon (*Cucumis melo* L.), watermelon (*Citrullus lanatus* L.), and squash (*Cucurbita* spp.). These crops are widely cultivated for fresh consumption and processing and contribute significantly to global food production systems [1]. However, cucurbit species are highly susceptible to a broad spectrum of viral pathogens, which constitute one of the major constraints on yield, fruit quality, and marketability [2]. Among these, cucumber green mottle mosaic virus (CGMMV), cucumber mosaic virus (CMV), and zucchini yellow mosaic virus (ZYMV) are particularly destructive, inducing symptoms such as mosaic patterns, leaf deformation, stunted growth, and fruit malformation, ultimately resulting in substantial economic losses worldwide [3,4,5].

Plants have evolved sophisticated antiviral defense mechanisms, among which RNA silencing represents a central and evolutionarily conserved strategy [6]. This process is mediated by small RNAs, including small interfering RNAs (siRNAs) and microRNAs (miRNAs), which regulate gene expression at the post-transcriptional level through mRNA cleavage or translational repression [7]. In plants, miRNAs are typically 20–24 nucleotides in length and are generated through a well-defined biogenesis pathway involving DICER-LIKE1 (DCL1), ARGONAUTE (AGO) proteins, and RNA-induced silencing complexes (RISC) [8,9,10,11]. Beyond their fundamental roles in plant development and physiology, miRNAs have emerged as key regulators of responses to biotic stresses, including viral infections [12,13,14].

During virus infection, host miRNA expression profiles undergo extensive reprogramming, leading to the modulation of genes involved in immune responses, hormone signaling, and developmental pathways, as demonstrated in several studies on cucumber and melon [15,16,17]. Concurrently, plant viruses have evolved counter-defense strategies by encoding viral suppressors of RNA silencing (VSRs), such as CMV 2b protein and HC-Pro from the potyvirus family, which disrupt the host’s RNA silencing machinery by targeting Argonaute proteins or binding small RNAs, thereby attenuating antiviral responses [18,19,20,21,22,23,24]. This presents a complex regulatory landscape in which miRNAs act both as central components of host defense and targets of viral manipulation.

In recent years, increasing evidence has demonstrated that plant antiviral responses cannot be explained by individual defense genes alone. Instead, resistance emerges from highly interconnected regulatory networks integrating miRNAs, transcription factors, hormone signaling, RNA silencing components, and other classes of non-coding RNAs. This systems-level perspective has substantially changed our understanding of plant–virus interactions and highlights miRNAs as central regulatory hubs rather than isolated gene regulators.

Recent high-throughput sequencing approaches have enabled the identification of numerous conserved and novel miRNAs responsive to viral infection in cucurbit crops, primarily in cucumber and melon. Key miRNA families, including miR156, miR159, miR168, miR169, miR171 and miR172, have been implicated in the regulation of transcription factors, resistance-related genes, and signaling pathways associated with plant immunity [15,16,17]. Other classes of non-coding RNAs, such as long non-coding RNAs (lncRNAs) and circular RNAs (circRNAs), may act as precursors and target mimics of miRNAs, forming a multilayered network [25,26,27,28,29,30]. However, this level of regulatory complexity remains largely unexplored in Cucurbitaceae. Although high-throughput sequencing technologies have enabled the discovery of numerous virus-responsive miRNAs and predicted regulatory interactions, functional characterization has progressed considerably more slowly. Consequently, much of the current knowledge is based on computational predictions and expression profiling, whereas experimentally validated miRNA–target interactions remain relatively limited. Bridging this gap represents one of the major challenges in understanding miRNA-mediated antiviral defense.

Despite these advances, several critical gaps remain. Current knowledge is fragmented and often limited to individual species, hindering a comprehensive understanding of conserved and species-specific miRNA-mediated responses across the Cucurbitaceae family. Most studies rely on expression profiling, while functional validation of miRNA–target interactions and their biological significance in antiviral defense remains insufficient. Moreover, although numerous virus-responsive miRNAs have been identified, the strength of evidence supporting individual miRNA–target interactions varies considerably among studies. Distinguishing computational predictions from experimentally validated interactions is therefore essential for accurately interpreting the current state of knowledge. Finally, the integration of miRNA pathways with broader regulatory networks, including other non-coding RNAs and hormone signaling pathways, is still poorly understood, particularly under field-relevant conditions. Therefore, this review aims to provide a comprehensive and integrative overview of miRNA-guided antiviral defense mechanisms in Cucurbitaceae. We focus on (i) the molecular interactions between miRNAs and viral factors, (ii) the role of miRNAs in shaping host responses to infection, (iii) the emerging concept of regulatory networks and cross-talk involving multiple classes of non-coding RNAs, and (iv) the potential application of miRNA-based strategies for improving viral resistance. By synthesizing current knowledge and identifying key research gaps, this review highlights miRNA-centered regulatory networks as a promising foundation for next-generation crop protection strategies. In addition, we emphasize the importance of integrating multi-omics datasets with functional validation approaches to translate rapidly accumulating sequencing data into mechanistic insights and practical applications for crop improvement.

## 2. Viral Threats in Cucurbitaceae

Viruses are among the major threats to crop production worldwide [31,32,33]. To date, over 2025 plant-infecting virus species from 73 genera and 49 families have been documented [33,34]. Plant viruses can be transmitted to new susceptible hosts horizontally (i.e., between hosts regardless of descent) and/or vertically (i.e., from parents to the offspring), with the former a key characteristic for pathogen fitness [35]. A database on the modes of transmission and vectors of over 1600 plant viruses has been created recently, which brings together a century of research in plant virology [36].

Viral pathogen, an obligatory intracellular parasite, relies on living cells for multiplication and utilizes the host translation machinery for translation of viral proteins [31,37,38]. Virus particles, or so-called virions, are the transmissible form of a virus. The virions consist only of genomic nucleic acid and protein, with the protein creating a protective coat around the nucleic acid [38]. Plant viruses enter their host cells only through wounds, e.g., made mechanically or by vectors, among other ways. The first intact virions may appear in plant cells approximately 10 h after infection [38]. After entering the plants, viruses move from one cell to adjacent cells through the plasmodesmatal channels and replicate in such cells. In leaf parenchyma cells, the virus moves approximately 1 mm, or 8-10 cells per day; most viruses require over 2–5 days to move out of an inoculated leaf [38]. After reaching the phloem, the virus moves fast toward apical meristems or other food-utilizing parts of the plant and spreads systemically throughout the plant; within an infected plant, the virus reenters the parenchyma cells adjacent to the phloem through plasmodesmata [38]. The viral infection cycle continues by transmission to new hosts via different mechanisms [37]. Infections caused by vector-transmitted viruses are polycyclic, e.g., for aphid-transmitted viruses the number of disease cycles per season can be over 10–20 [38].

The number of viruses capable of infecting cucurbits is increasing. Decades ago, there were at least 59 well-studied viruses of the major plant virus groups affecting cucurbits worldwide [2]. To date, up to 90 viruses can infect cucurbits in natural conditions [39]. The main viruses currently threatening cucumber and other cucurbits, e.g., melon, watermelon and squash, include CMV, CGMMV, ZYMV and WMV (Table 1). Many recent reviews and research findings indicated spreading these viruses in cucurbits crop in different regions and continents worldwide, e.g., Spain, Mediterranean region, India, Oklahoma, Georgia, Southeast China, Saudi Arabia, Caribbean region, Australia, Marmara region, Jordan, Egypt, Turkey, Argentina, Syria, Croatia, the U.S., Azerbaijan, Pakistan [33,40,41,42,43,44,45,46,47,48,49,50,51,52,53,54,55,56,57,58,59,60,61,62,63,64,65,66,67,68,69,70,71,72,73,74,75]. There are several ways to control cucurbit viruses, e.g., using healthy seeds, vector control, and genetic control using resistant cultivars, cross protection [2,67,76,77,78,79,80,81].

Despite the importance of these management strategies, their effectiveness is often challenged by the rapid evolution of viral populations, the emergence of new strains, and the complexity of host–virus interactions. Consequently, increasing attention has been directed toward endogenous molecular defense mechanisms, particularly RNA silencing and miRNA-mediated regulation, as complementary approaches for improving durable virus resistance.

For non-persistent transmitted viruses, e.g., the aphid-transmitted ZYMV, WMV, and CMV, where vector control is challenging due to very short periods between acquisition and transmission, genetic control provides alternative management [67,76,77,78,79]. For example, sources of resistance against WMV can be found in local melon and squash cultivars and wild desert bitter watermelon [79], or in watermelon cultivars against ZYMV [67]. In a recent study, a bivalent plant viral vaccine, i.e., an attenuated recombinant virus ZAC-MYnp, conferred protection against ZYMV and an orthotospovirus [81]. This is a good example of mild strain-mediated cross-protection against taxonomically distant viruses in cucurbit hosts [81].

CMV is among the latest “Plant Virus Top Ten” list affecting the most important food crops [38]. A recent commentary re-enforced that CMV is a silent threat to global cucurbit production [63]. The author points out that the impact of CMV can be mitigated through cultural practices, vector control, and using resistant cultivars [63]. For example, the melon gene, i.e., *CmVPS41*, conferred resistance to CMV by preventing the phloem entry of the virus [82]. Joshi et al. [72] suggested that cleaning weeds from cucurbit field borders is essential for controlling the source of infectious material, because many weeds are asymptomatic hosts, and act as reservoirs for CMV [72].

The seed- and contact-transmitted CGMMV can spread over long distances without a vector; its particle is very stable, which makes the disease cycle of this virus very complex [77,83]. CGMMV can persist in the absence of growing cucurbit crops through contaminated cucurbit plants and seeds, weed hosts, and its virions can remain infectious in soil [3]. Shargil et al. [84] demonstrated that the pathway of CGMMV to infect seed coat may have originated from the maternal flower, which may help to develop concrete seed disinfection strategies to prevent CGMMV spreading [84]. Ellouze et al. [85] reported the control of CGMMV using agricultural disinfectants and resistant cucumber cultivars [85]. Lovelock et al. [86] highlighted the importance of proper weed management to effectively manage CGMMV [86]. Liu et al. [87] and Crespo et al. [88] identified potential sources of resistance against CGMMV in watermelons and cucumbers [87,88]. Cross-protection with attenuated ZYMV strains has been used on cucurbit crops against CGMMV under field conditions [3,55,89,90].

CMV, CGMMV, ZYMV and WMV affect production of cucurbits worldwide by reducing fruit yield and quality, resulting in significant economic loss (Table 1).

**Table 1:** Main viruses currently threatening cucumber, melon, watermelon and squash production worldwide.

Virus Name Abbreviation	Virus Species/ Genus/ Family	Virus Genome/Strain Group	Transmission	Symptom	Impact on Yield/Fruit Quality	References
CMV	*Cucumovirus CMV*/*Cucumovirus*/*Bromoviridae*	Positive sense ssRNA: RNA1, RNA2, RNA3, subgenomic RNA4, satRNA/ IA IB II	Aphid (non-persistent) Plant dodder Seed Mechanical	*Cucumber*: YGM on leaves and fruits; MOT, DEF and ENA of fruits; WP. *Other cucurbits*: MOT, M, DC, YLM on leaves; flower ABN; M, MAL fruits, RFS; PST.	Reduced photosynthesis; up to 50% yield losses on cucurbits. Reduce fruit quality making the crop less marketable	[41,42,63,72,82,91]
CGMMV	*Tobamovirus viridimaculae*/*Tobamovirus*/*Virgaviridae*	Positive sense ssRNA (monopartite)/ Cluster (I) Cluster (II)	Seed Mechanical Contact Pollen cucumber leaf beetle Honeybee	*Cucumber*: DEF, M, MOTM, interveinal CHL, BLI, BYIs-DGS in leaves, DIST, M in fruits, MOT of leaves and fruit, COIP. *Melon*: M in leaves; MAL, MOT, SN of fruits. *Watermelon*: WP, NL on peduncle, M, MOT, CHL, RLS on leaves, SPOP, ROT, DISC BFD of flesh, SSI, PST. *Squash*: NEC, DISC in internal fruit.	Trade restrictions, economic losses due to inability to re-plant for many months, over 50% yield losses in cucumber and watermelon, fruit symptoms affecting marketable yield or resulting in unmarketable.	[3,73,74,75,83,84,86,87,88,92,93,94,95,96]
ZYMV	*Potyvirus cucurbitaflavitesselati*/*Potyvirus*/ *Potyviridae*	Positive sense ssRNA (monopartite)/ A (I–IV) B (I–II) C	Aphid (non-persistent) Seed Mechanical Contact	*Cucumber*: VCL, DEF, M with BUB, BLI, SE, ENA, YLM, LP on leaves; ENA, M, NC, severe DEF on fruits; Mild SPO, severe M on fruit skin; PST. *Other cucurbits*: M, DEF, CHL, MAL, RLS, SHOEST, RLFP, NCHL on leaves, MAL and SDISC on fruits.	Impaired photochemistry, photoprotection and light interception; enhanced photoinhibition; up to 100% yield loss. Reduction in marketable fruits.	[5,41,42,50,53,66,70,71,97,98,99,100,101]
WMV	*Potyvirus citrulli*/*Potyvirus*/ *Potyviridae*	Positive sense ssRNA (monopartite)/ G1 (CL) G2 G3 (EM)	Aphid (non-persistent) Mechanical	*General symptoms*: M, CHL, VCL, VBAN, DEF, BLI, RLS, SHRI in leaves, DISC, DIST, IRRIP, BUB, SPO in fruits, DEF of leaves and fruits, PST	Reduction of yield 35% in mixed infection with WMV, ZYMV and CMV. Reduction in marketable fruits.	[46,55,56,60,65,71,102,103,104,105,106,107,108]

Virus name: CMV—cucumber mosaic virus, CGMMV—cucumber green mottle mosaic virus, ZYMV—zucchini yellow mosaic virus, WMV—watermelon mosaic virus (WMV). Virus genome: ssRNA—single-stranded RNA, Sat RNA—Satellite RNA. Strain Gropp: CL—Classic, EM—Emergent. Symptom: ABN—abnormalities, BFD—a pulp deterioration called blood flesh disease, BLI—blistering, BUB—bubbling, BYIs-DGS—bright yellow islands (BYIs) surrounded by dark green surrounding (DGS) tissues in leaves, CHL—chlorosis, COIP—collapse of infected plants, DC—distorted and curled, DEF—deformation, DISC—discoloration, DIST—distortion, ENA—enation, IRRIP—irregular ripening, LP—leaf pallor, M—mosaic, MAL—malformation, MOT—mottling, MOTM—mottle-mosaic on leaves, NC—necrotic cracks, NCHL—necrotic and chlorotic lesions on leaves, NEC—necrosis, NL—necrotic lesions, PST—plant stunting, RFS—reduced fruit set, RLS—reduced leaf size, RLFP—rupture of the leaves in a filamentous pattern, ROT—rotting, SDISC—surface discoloration, SE—sawn edges, SHOEST—characteristic “shoestring” leaf phenotype, SHRI—shrinkage, SN—surface netting, SPO—spotting, SPON—spongy, SSI—shorter stem internodes, VBAN—vein banding, VCL—vein clearing, WP—wilting of plant, YGM—yellow or green mosaic, YLM—yellowing and leaf malformation.

Collectively, the major cucurbit viruses differ in their taxonomy, transmission routes, and symptom development; however, they all induce extensive reprogramming of host gene expression and RNA silencing pathways. Understanding how miRNA-mediated regulatory networks respond to these viral infections is therefore essential for deciphering the molecular basis of antiviral defense in Cucurbitaceae. The following section summarizes the molecular basis of RNA silencing and discusses how virus-responsive miRNAs contribute to the establishment, modulation, and fine-tuning of antiviral defense mechanisms in Cucurbitaceae.

## 3. RNA Silencing and miRNA–Virus Interactions

RNA silencing represents one of the most fundamental antiviral defense strategies in plants, providing a sequence-specific mechanism for the recognition and elimination of invasive nucleic acids. The central role of RNA silencing in plant–virus interactions has been documented in both classical and recent studies [6]. This system operates through DCL-processed small RNA molecules, primarily siRNAs and miRNAs, which guide AGO-containing effector complexes to complementary RNA targets, resulting in transcript cleavage or translational inhibition [6,7,109]. Among protein families involved in RNA silencing are also RNA-dependent RNA Polymerase (RDR), an amplifier of the silencing effect, and Suppressor of Gene Silencing (SGS), which produces secondary siRNAs and reinforces the silencing process [109]. While siRNAs are typically derived directly from viral replication intermediates and act as primary antiviral effectors, miRNAs play a more nuanced role by modulating endogenous gene expression programs that shape the host’s response to infection [8,14].

### 3.1. Core Components of RNA Silencing and miRNA Biogenesis

In plants, miRNAs originate from endogenous *MIR* genes transcribed by RNA polymerase II into several hundred nucleotide-long primary miRNA transcripts (pri-miRNAs), featuring one or more miRNA stem loops. These pri-miRNAs are subsequently processed in the nucleus by DCL1 into precursors (pre-miRNAs), and then into mature miRNA duplexes. These duplexes are methylated and exported to the cytoplasm, where one strand is selectively incorporated into an AGO protein to form RISC [8,9,10,11]. The AGO protein then guides mature miRNA to target transcripts with near-perfect complementarity, most commonly resulting in mRNA cleavage.

This canonical pathway is closely integrated with antiviral RNA silencing. In parallel to miRNA production, viral double-stranded RNA is processed by DCL enzymes into virus-derived siRNAs (vsiRNAs), which are loaded into AGO proteins and direct the degradation of viral genomes [6,110]. The convergence of these pathways at the level of AGO proteins establishes a shared regulatory platform in which endogenous and antiviral silencing mechanisms are functionally intertwined.

### 3.2. Viral Suppressors of RNA Silencing

The efficiency of RNA silencing as an antiviral barrier has driven the evolution of viral countermeasures collectively known as VSRs. These proteins target several key steps of the RNA silencing pathway. A well-characterized example is the 2b protein encoded by CMV, which directly interacts with AGO1 and inhibits its slicer activity, thereby compromising both siRNA- and miRNA-mediated silencing pathways [18,21,22]. In addition, 2b can bind small RNAs, further limiting their availability for incorporation into RISC.

Similarly, HC-Pro, a multifunctional protein encoded by potyviruses such as ZYMV, acts primarily by sequestering small RNAs and interfering with their stabilization and loading into AGO proteins [19,20,23,24]. Although these suppressors differ in their modes of action, their overall effect is the attenuation of RNA silencing, allowing viral genomes to evade degradation and establish systemic infection. Recent reviews further highlight that VSRs not only suppress antiviral pathways but also broadly reprogram host regulatory networks, including miRNA-dependent gene regulation [21,22,23,24,111]. The ability of viral suppressors to interfere simultaneously with antiviral defense and endogenous developmental pathways further illustrates the central regulatory role of miRNAs during plant–virus interactions.

### 3.3. miRNA-Mediated Regulation During Viral Infection

The interaction between miRNAs and plant viruses is inherently bidirectional. On the one hand, viral infection triggers substantial changes in host miRNA expression profiles, leading to the reprogramming of regulatory pathways associated with immunity, development, and hormone signaling [15,16,17]. On the other hand, viruses actively manipulate these pathways to create a cellular environment conducive to their replication, as increasingly recognized in recent integrative studies [112,113]. Numerous miRNAs have been implicated in the regulation of transcription factors and signaling components involved in antiviral responses. These include members of the *MYB*, *SPL*, and *bZIP* families, which act downstream of miRNA-mediated regulation to coordinate defense activation and developmental processes [15,16,17]. The resulting regulatory network is highly dynamic, reflecting the integration of multiple signaling inputs during infection and environmental stress [113].

Recent advances indicate that miRNA-mediated regulation in plant–virus interactions extends beyond simple one-to-one relationships between miRNAs and their targets. Instead, these interactions form part of complex regulatory networks involving multiple layers of control. miRNAs can regulate multiple genes simultaneously, while individual transcripts may be targeted by several miRNAs, creating a highly interconnected system [114,115]. Furthermore, other classes of non-coding RNAs, including lncRNAs and circRNAs, participate in these networks by modulating miRNA activity through competitive endogenous RNA (ceRNA) mechanisms [22,25,26,27,28,30]. These regulatory cross-talk between different RNA classes may play a role in fine-tuning gene expression during viral infection. Although these regulatory networks are increasingly supported by transcriptomic and small RNA sequencing studies, many of the proposed miRNA–target interactions still await direct experimental validation, highlighting an important gap between network prediction and mechanistic understanding.

RNA silencing and miRNA-mediated regulation are components of an integrated regulatory network, which interact in response to factors associated with viral infection. Understanding this complexity is essential for deciphering the molecular basis of antiviral defense and for the development of RNA-based strategies aimed at improving crop resilience. These molecular mechanisms provide the conceptual framework for understanding how individual miRNA families contribute to antiviral responses against specific cucurbit viruses, as discussed in the following section.

## 4. The Role of miRNAs in the Response to Main Viral Threats of the Cucurbitaceae Family

MicroRNAs play a key role in shaping plant responses to viral infection by modulating gene expression at multiple regulatory levels. In Cucurbitaceae, recent advances in high-throughput sequencing and transcriptomic analyses have revealed extensive reprogramming of miRNA expression in response to viral pathogens. Although the impact of small RNA expression on viral infection has been extensively studied for several viruses [15,116,117,118,119], miRNA expression profiles have been characterized only for CGMMV and CMV [15,16,17].

The studies included in this review, conducted primarily on cucumber and melon but also on other crop species affected by these viruses, have identified both conserved and species-specific miRNAs that contribute to antiviral defense as well as to symptom development [15,16,17]. It is worth noting that several families of miRNAs have only been reported in plants from other families, such as *Arabidopsis*, tobacco, and tomato, which may be due both to the limited number of studies on this topic, and to differences in how individual species respond to viruses. The collected data on the virus-responsive miRNAs are summarized in Table 2.

As summarized in Table 2, most currently reported miRNA–target interactions in Cucurbitaceae are supported primarily by computational prediction or expression-based evidence, whereas experimentally validated interactions remain relatively scarce. This highlights the need for further functional studies to establish causal regulatory relationships.

### 4.1. Broad Reprogramming of miRNA Expression During Viral Infection

Viral infection induces significant alterations in the abundance, composition, and size distribution of small RNAs in cucurbit species. For instance, CGMMV infection in cucumber and melon has been associated with a tendency toward shorter sequences within many found miRNA families, suggesting that the infection itself was responsible for the shortening of these sequences [15,17]. Genome-wide analyses have identified numerous conserved and novel miRNAs that are differentially expressed during infection, indicating large-scale reorganization of post-transcriptional regulatory networks [16,17]. These results suggest that viral infection triggers a broad remodeling of the small RNA landscape, which is likely essential for the coordination of miRNA-mediated antiviral defense.

### 4.2. miRNA Targets and Functional Implications

The biological function of miRNAs depends largely on their target genes. Virus-responsive miRNAs in cucurbits are known to interact with transcription factors such as *MYB*, *bZIP*, *SPL*, and *NAC* families, which are involved in the regulation of defense responses, hormonal signaling and plant development [16,17]. In addition, subsequent studies have identified miRNA target genes involved in processes such as cell wall metabolism, transport processes and signaling pathways (Table 2). This implies that miRNA regulation of viral infection encompasses both the response to viral pathogens and general changes resulting from infection. Importantly, most target genes were identified in studies of specific plant species, regardless of their ubiquity, highlighting a critical gap in the current knowledge that hinders an accurate assessment of the role of miRNAs in plant–virus interactions. Furthermore, much of the data on expression and functions of these miRNAs still requires validation, meaning that some of the information presented should be considered speculative.

### 4.3. Key Conserved miRNA Families Involved in Antiviral Responses

**Figure 1:**
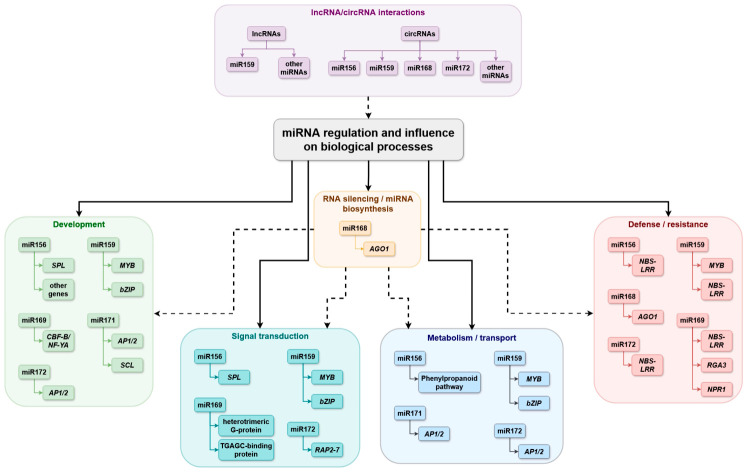
Selected key miRNA families involved in miRNA regulation and influence on plant’s biological processes—miR156, miR159, miR168, miR169 miR171, miR172—and their target genes involved in the plant response to CGMMV and CMV, grouped by the type of process they regulate. Dashed lines represent the interactions between miRNAs and other non-coding RNAs, as well as the influence of miRNA biosynthesis pathway on other processes regulated by miRNA.

Several conserved miRNA families have been identified as key regulators of the viral infection response in cucurbits. These miRNAs influence the expression of numerous target genes involved in a range of crucial plant processes, including regulation of growth and development, signal transduction via factors such as phytohormones, responses to abiotic and biotic stresses, regulation of resistance (R) genes and infection responses, transport, and metabolism. Key miRNA families, including miR156, miR159, miR168, miR169, miR171 and miR172, are involved in the regulation of many of these processes, serving as central components of a larger regulatory network associated with the infection response (Figure 1).

miR156 is a key regulator for developmental timing and phase transitions. In cucumber infected with CGMMV, miR156 is upregulated, which leads to repression of *SPL* transcription factors and, consequently, delayed flowering, leaf deformation, and reduced fruit production. Furthermore, it also influences the expression of miR172, another important regulator of this pathway, which acts downstream of miR156 [17]. Other target genes of miR156 may also affect processes such as pollen maturation, flowering time, signal transduction, phenylpropanoid synthesis, and microbial defense in response to CGMMV [15,17]. The versatility of miR156’s role in regulating viral infection suggests that it is essential for the proper functioning of defense mechanisms. Similarly, it has been shown that *SPL* genes influence tomato flowering time in response to CMV [122,123]. In cucumber, this miRNA was also associated with genes from the *NBS-LRR* family, a crucial class of resistance genes, and was responsible for downregulating the expression of these genes in absence of infection [121].

miR159 is one of the most important miRNAs in viral response. Its differential expression regulates a variety of processes related to disease resistance and modulation of stress tolerance. This miRNA primarily targets transcription factors from the *MYB* family, which are associated with hormonal signaling pathways, as identified in studies on cucumber, *Arabidopsis*, tobacco and tomato [16,122,125,127,128]. During CGMMV infection, miR159 is associated mainly with the regulation of stress tolerance, playing a potential role in mediating plant–virus interactions [16,120]. The presence of a salicylic acid (SA)-binding target protein also suggests that the viral response in cucumber may be linked to the SA signaling pathway. It has been shown that the altered expression of miR159 during CMV infection is responsible for the symptoms caused by severe CMV strains in *Arabidopsis* and *N. benthamiana*, including stunted growth and plant deformation [125,126]. In tomato, CMV infection causes changes in the development of leaves, flowers and fruits, particularly in the regulation of flowering time [122,128]. One of the *MYB* family targets found in tomato, *GAMYB-like1*, is regulated by gibberellic acid (GA), further confirming the link between miR159 and hormonal signaling pathways. In tobacco, target genes have been assigned to *MYB* and *bZIP* families, which play roles in plant development, stress responses, hormone signaling, and metabolic regulation [128]. Infection of cucumber also unveiled that miR159 can target *NBS-LRR* genes, mediating a response to CMV [16]. Its role in both the spread and mitigation of the disease underscores the importance of this miRNA in the course of viral infections.

miR168, a conserved miRNA involved in the self-regulation of miRNA biosynthesis pathway through cleavage by AGO1 proteins, plays a universal role in the induction of antiviral responses, a role that remains consistent regardless of plant species or virus [127]. Several studies on the CMV response in *Arabidopsis*, tobacco and tomato (Table 2) have highlighted its role in plant defense mechanism, responsible for increasing miRNA levels during infection when it is downregulated, as well as for inhibiting antiviral RNA silencing when its levels are increased. This affects the expression of all miRNAs, making miR168 the primary mediator of viral response. miR168 also forms a regulatory network with *AGO1*, miR403 and its target gene *AGO2*, which is responsible for symptom development and induction of anti-viral RNA silencing in CMV-infected *Arabidopsis* plants [119]. Interestingly, although the miR168–AGO1 interaction has been demonstrated in response to CMV infection, data regarding the presence of this miRNA in CGMMV are scarce. It has been found to regulate the stress response in watermelon [120], but none of the CGMMV studies included in this review demonstrated a role for miR168 in the self-regulation of disease-responsive miRNAs. Since miR168 is a central regulator of AGO1 homeostasis, its induction is frequently associated with perturbations of the AGO1 pathway. Unlike CMV, which encodes the well-characterized 2b suppressor that directly interacts with AGO1 and inhibits AGO1-mediated RNA silencing, CGMMV, a member of the genus Tobamovirus, employs distinct mechanisms to modulate host defense responses. CGMMV encodes the 129K/186K replication proteins, which possess RNA silencing suppressor activity; however, unlike CMV 2b, they have not been shown to directly interact with AGO1. The absence of significant miR168 alterations during CGMMV infection may therefore reflect differences in viral suppressor activity and suggest that perturbation of AGO1-centered regulatory circuits is not a universal feature of cucurbit virus infections. Nevertheless, this hypothesis requires direct experimental validation [138,139].

miR169, one of the major miRNAs participating in plant immunity, targets numerous genes involved in initiating the defense response under stress conditions. Remarkably, it has been shown that this miRNA regulates its target genes primarily by inhibiting translation, rather than through the cleavage typical of plant miRNAs [16]. The targets of miR169 in CGMMV-infected cucumber were identified as genes encoding a heterotrimeric G-protein and an RGA3-like protein, which are involved in triggering the defense system that inhibits pathogen growth, as well as in phytohormone signal transduction [16,17]. CMV infection has also been linked to disease-associated target genes, with *NBS-LRR* genes identified in cucumber and the *NPR1* gene in tomato [121,123]. It has been suggested that miR169 target genes in tomato play a role in the initiation of the defense response, but also in pathogen recognition, SA signal transduction, cross-talk of salicylic- and jasmonate-dependent pathways, and even in cytoskeleton assembly and organization [123]. Effects on embryo morphogenesis and cell differentiation have also been observed in tobacco [127]. In summary, miR169 appears to be a miRNA strongly involved in the formation of plant antiviral defense mechanisms.

miR171 regulates the activity of transcription factors involved in developmental processes. Importantly, it was found that in cucumber infected by CGMMV, miR171 interacts with *AP1/2* genes, which control floral organ differentiation, in a process co-regulated with miR172 [17]. Furthermore, two other genes identified in the study were associated with processes such as transcription regulation, growth, and, most notably, phloem transport. It is suggested that miR171 may be involved in the transport of CGMMV within the plant [17,132]. Based on studies of tobacco, tomato, and hot pepper, it was found that miR171 influences CMV infection through *SCL* genes—transcription factors involved in the regulation of flowering time, as well as shoot and root development [122,127,133]. It therefore appears that infection affects these processes, revealing a link between viruses and plant organization.

miR172 is an important regulator of phase transitions, influencing developmental changes through interactions with other miRNAs, such as miR156. Similar to miR171, it is also involved in regulating CGMMV transport within cucumbers via *AP1/2* genes, as well as in regulating developmental stages, growth and flower organ differentiation through *AP1/2* and other target genes. Additionally, it is also involved in responses to stress factors and phytohormone signals, with a notable target being an ethylene-responsive *RAP2-7-like* transcription factor [16,17]. *AP2* genes are also involved in response to CMV infection and may induce phenotypic changes in tomato as a result [122,123]. In cucumber, miR172 was among miRNA found to target *NBS-LRR* genes, demonstrating its role in regulating plant resistance to CMV [121]. This suggests that miR172 is involved in both virus-induced developmental changes and the response to viral infection.

miR156, miR159, miR168, miR169, miR171, and miR172 are characterized in defense response to CGMMV and CMV of many plant species, including several Cucurbitaceae family members, and they belong to conserved miRNA families present in most plant species. These miRNAs regulate a broad range of plant defense-related processes, which can be categorized into essential types of regulations such as development, defense and resistance, metabolism and transport, and signal transduction (Figure 1). They perform an important, non-specific role in mediating the plant response to the CGMMV or CMV, which is signified by the association of viral infections with changes in their expression profiles across many studies. Therefore, the presented miRNAs appear to be significant components of plant defense against these viruses.

### 4.4. Other Significant miRNAs Involved in Antiviral Responses

Although the processes related to viral infection are most associated with a few multifunctional miRNAs, the networks they form are further regulated by several other miRNAs that also participate in the precise modulation of the defense response. This chapter aims to highlight additional aspects of how miRNAs affect infected plants.

miR160 is implicated in the regulation of hormonal signal transduction. In CGMMV infection of cucumber, it has been found to target a heterotrimeric G-protein, in similar manner to miR169, while CMV has been associated with auxin-responsive factors *ARF10* and *ARF17* in tobacco and tomato [17,122,123,127]. Furthermore, miR160 is believed to cause the production of seedless fruit in tomato.

Another family involved in regulating the miRNA biosynthesis pathway—primarily by interacting with the DCL1 protein—miR162 also plays a role in regulation of resistance genes. It has been shown that this miRNA targets *LRR* and *RGA* families in watermelon, as well as NBS-LRR proteins during cucumber infection. It is involved in the self-regulation of miRNA levels and in modulating plant resistance to viruses with its expression increasing following CMV infection in tomato and decreasing in tobacco [15,121,122,127,129].

CMV infection in tobacco and tomato identified miR164 as regulator of several plant developmental processes through *NAC1* and *CUC2* genes, including the several processes in the formation of meristems, leaf development, and the control of the proper functioning of these processes. A change in the expression of this miRNA causes leaflets to fuse and affects leaf serration. Moreover, it may also be involved in the viral defense mechanism of these plants [127,128,129,130,131]. Currently, there are no data demonstrating the effect of miR164 on the progression of CGMMV infection, nor are there any studies showing this miRNA’s influence on infection in cucurbits.

Like miR164, miRNAs from the miR165 and miR166 families participate in developmental processes triggered by CMV infection by regulating meristem formation via the HD-ZIP III family proteins, and they also play a role in hormonal signaling. It has been shown that inhibiting the activity of these miRNAs induces disease symptoms, leading to stunted growth and deformities of leaves and flowers. Although miR165/166 were detected in most plants studied for the effects of CMV infection on miRNAs [123,126,127,128,129,130], their role in viral infection of cucurbits has not yet been demonstrated. It has been found that members of miR165/166 respond to CGMMV infection in watermelon [15], but their presumed function has not yet been determined.

It has been found that miR167 acts as a regulator of flower maturation, leaf development, and fruit set initiation during CMV infection. Its target genes include the PP2C protein in tobacco and the auxin-responsive factors *ARF6* and *ARF8* in tomato [122,123,127,128], indicating that this response is regulated by phytohormonal signals. Interestingly, this contradicts the results of an earlier study on tomato, in which differential expression of miR167 was not observed [129], which may suggest the existence of other factors affecting its expression. Furthermore, miR167 has been identified in the stress response to CGMMV in watermelon [120].

miR319 influences the course of infections caused by CGMMV and CMV by interacting with genes from the *TCP* family. These genes are involved in regulating leaf morphology, cell cycle, and meristem function. An interesting aspect of miR319 is that it regulates expression of miR396—a miRNA that targets growth factors and *NBS-LRR* genes in cucumber and tomato [17,121,123,129,130,133,134,135]. miR319 and miR396 are responsible for regulating plant development and its response to diseases. A change in expression of miR319-miR396 module causes leaf malformation and stunted plant growth, which can result in yield losses [17,134,135]. Furthermore, it has been suggested that they may play a role in the defense response to CMV infection in cucumber, hot pepper, and tomato [121,123,129,130,133]. It has been found that miR396 also regulates the stress response in watermelon [120].

miR390 is another miRNA targeting resistance gene families, such as *NBS-LRR* and *RGA*, as well as proteins involved in the plant defense mechanisms. Infection of cucumber with CGMMV causes delayed flowering and reduced fruit yield, which is attributed to the regulation of miR390 [15,17,121]. In tomato, CMV infection is associated with signal transduction, regulation of plant development, and a suggested potential role in regulating chlorophyll and heme levels [123,128].

miR393 is a conserved family of miRNAs that plays a significant role in the stress response of many crop plants. Its expression has been shown to be altered in response to viral infection in soybean [140], rice [141] and tomato [142]. miR393 has been found to regulate the auxin signaling pathway in CGMMV-infected watermelon by targeting genes coding pectinesterase and its inhibitors. It has also been linked to host–cell receptor recognition, which may influence plant immunity [15]. miR393 may also regulate plant development in response to CMV infection in *Arabidopsis*, but its exact function has not yet been demonstrated [124].

miR403 is a component of the miRNA biosynthesis pathway in *Arabidopsis* during CMV infection, directly regulated by the miR168–AGO1 complex, which in turn interacts with the AGO2 protein. miR403 has been found to perform a role in the induction of antiviral RNA silencing following infection, caused by a decrease in *AGO1* expression [119].

miR408 is involved in a range of processes related to signal transduction, cell wall modifications, and copper transport. In cucumber, it regulates disease resistance by targeting *heterotrimeric G-protein* genes [17], while in watermelon it mitigates the effects of viral attacks by regulating *laccase 3* gene [15]. It has also been found that, during the response to CMV in tomato, it influences *copper-transporting ATPase PAA2* gene [123].

miR482 is involved in pathogen recognition and the initiation of the defense response by interacting with *NBS-LRR* resistance genes in hot pepper and tomato. This leads to a plant defense response and the regulation of plant development in response to disease [123,133]. miR482 has also been identified among stress-responsive genes in watermelon [120].

miR838 acts as a regulator of several pathways involved in plant resistance to pathogens. In cucumber, it has been linked to a CAD1-like protein, regulating an SA-mediated programmed cell death pathway during hypersensitivity responses [16]. In watermelon, it has been associated with auxin transporters, resistance genes from *LRR* and *RGA* families, as well as genes involved in phenylpropanoid biosynthesis pathway. Consequently, the plant regulates the expression of resistance genes and the levels of auxins and phenylpropanoids, leading to stress responses and antimicrobial activity [15].

**Figure 2:**
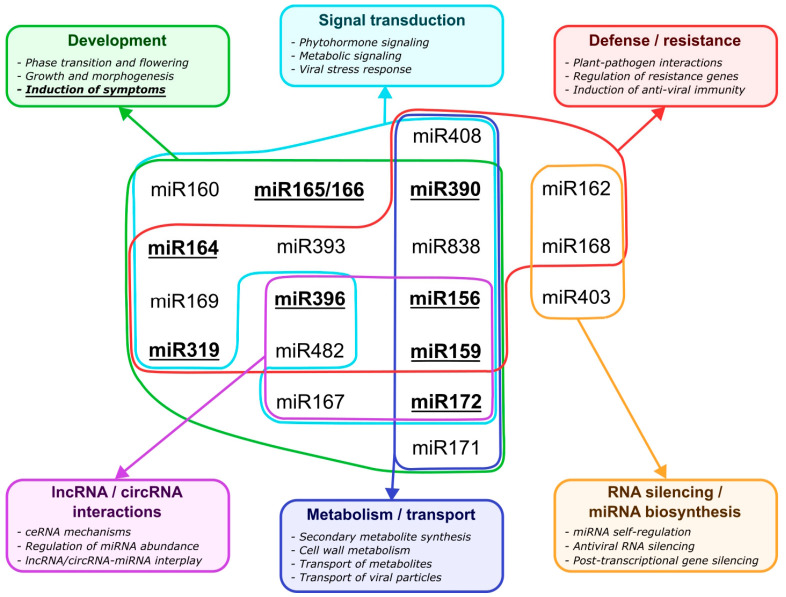
Multifunctional regulatory role of selected miRNA families that are important in response to viral diseases of Cucurbitaceae, grouped according to the types of processes they were proven to participate in: plant development (green), signal transduction (light blue), defense and resistance (red), lncRNA/circRNA interactions (purple), metabolism and transport (blue), and RNA silencing and miRNA biosynthesis (orange). miRNA families known to cause infection symptoms—miR156, miR159, miR164, miR165/166, miR172, miR319, miR390, miR396—are in bold and underscored.

Furthermore, several other miRNAs have also been shown to exhibit changed expression in response to CGMMV and CMV, as presented in Table 2. As such, the highlighted miRNAs are only a fragment of the larger regulatory network responsible for plant–virus interactions. These miRNAs represent important molecules connecting different branches of plant stress response type. Both conserved and non-conserved miRNAs contribute to an infection response specific to the plant species and their growth conditions, affecting several key types of processes by co-regulation (Figure 2). These processes and their miRNA-based interactions are the subject of the next section.

### 4.5. Co-Regulation of Plant Immunity Processes by miRNAs

The systemic response to plant pathogens mediated by miRNA is a complex regulatory process involving an intricate network comprising both conserved and species-specific miRNAs, their target genes, and other factors influencing their expression. These networks can regulate specific types of processes in a virus-affected plant, and thanks to the presence of conserved miRNAs regulating several networks, they form a coherent defense system. miRNAs are particularly involved in the regulation of signaling pathways, resistance genes, metabolite synthesis and transport, plant developmental processes, stress responses, and interactions with other molecules (Figure 2).

#### 4.5.1. Signal Transduction

The regulation of signal transduction is one of the most important ways in which a plant controls its response to its surrounding environment. For instance, the PP2C protein, involved in the regulation of development, is controlled by miR167 in tobacco and miR390 in tomato, in both cases during the response to CMV [123,127,128]. Similarly, infection of cucumber with CGMMV triggers the regulation of heterotrimeric G-proteins by miR160, miR169, and miR408 [17]. There is also a link to plant metabolism, as miRNAs associated with fatty acid signaling pathway—miR894, miR5303, miR8051 and miR8578—exhibited differential expression in watermelon following CGMMV infection [15].

Phytohormone signaling pathways are of particular importance as major signaling networks. The response to auxins, which is related to many developmental changes occurring within the plant, is regulated by several miRNA families. Infection with CGMMV in watermelon is associated with differential expression of miR393, miR838, miR1222, miR2655, miR8578, and miR9752, which are linked to both enhanced defense against the virus and the induction of disease symptoms [15]. Similarly, miR160 and miR167 influence the auxin response in CMV-infected tomato and tobacco, thereby affecting regulatory processes [122,123,127].

CGMMV infection appears to be regulated by ethylene in a manner specific to cucurbits. In cucumber, miR172 and a novel miRNA described by Liu et al., [17] target ethylene responsive transcription factors, mediating its signal transduction pathways [16,17]. In watermelon, miR1027, miR1028, and miR5741 were shown to regulate ethylene signaling. Furthermore, it was found that miR5741 also regulates cytokinin signaling, suggesting that this phytohormone may also be involved in plant defense against certain pathogens [15,143].

miR477, identified as a CGMMV-responsive miRNA in watermelon, is involved in pathways regulated by *DELLA* protein genes, which are related to GA, SA and JA, all of which play a role in regulating the plant immunity response [15]. Moreover, miR159 interacts with SA-binding proteins in CGMMV-affected cucumber [16], whilst miR169 regulates cross-talk between SA and JA in tomato [123]. This highlights the significance of phytohormone regulation during viral infection for the disease response, which is mediated by specific miRNAs.

#### 4.5.2. Plant Resistance and Viral Stress Response

It appears that the miRNA response to microbial attacks occurs at several regulatory levels mediated by numerous miRNAs that interact with one another. The most significant of those interactions involve resistance (R) genes, which are responsible for induction of plant resistance to pathogens. Infection of watermelons with CGMMV was associated with the regulation of genes from the *LRR* and *RGA* families, as well as defense-related proteins, via miR162, miR390, miR838 and miR1027. Another role of miR1027 is the regulation of host–cell receptor recognition, together with miR393, miR2873 and miR8578, with all these miRNAs being associated with pectinesterase activity. Furthermore, miR530 and miR5303 mediated plant immunity by regulating viral RNA replication [15].

Studies on the tomato’s response to CMV have shown that miR169, miR482, miR5301 and miR6022 are involved in the regulation of resistance genes, each of which targets a different R gene family [123,133]. Investigations of CMV-infected cucumber revealed that several conserved miRNA families target *NBS-LRR* genes, including miR156, miR157, miR159, miR162, miR169, miR172, miR390 and miR396 [121]. Another study on cucumber demonstrated that virus-responsive miRNAs form regulatory networks amongst themselves, the most significant of which involves miR159, miR838, miR854, miR5658 and a novel miRNA, csa-miRn6-3p [16].

miR159 is one of the miRNAs shown to induce symptoms in plants infected by severe CMV strains in plants, such as *Arabidopsis*, *N. benthamiana* and tomato [122,125,126,128]. miR854 and miR5658 mediate plant-pathogen interactions, influencing the disease response in cucumber [16]. The novel miRNA was found to regulate an *RGA* family gene and numerous other targets, which collectively regulate plant resistance to pathogens, growth and stress response, whilst simultaneously causing leaf mottling as a result of infection.

The results present the multifunctional response of the cucumber to viral attack, highlighting the dual nature of the miRNA response to viruses. A number of identified miRNAs, including key regulatory miRNAs such as miR159, are responsible for both the induction of plant immune systems against viruses and the manifestation of disease symptoms. This concept, which bridges the gap between susceptibility and immunity, requires further research to understand its complexities.

#### 4.5.3. Transport and Metabolism

Another important aspect of the interaction between miRNAs and viruses appears to be the transport of molecules within the plant and the regulation of metabolic pathways. It has been shown that miR171 and miR172 may be directly involved in transport of CGMMV particles in cucumber via the phloem, through the regulation of four genes, including the *AP1/2* transcription factors [17,132]. In addition to viral transmission, miRNAs have also been shown to regulate intracellular transport in response to CGMMV through ABC transporters in watermelon, mediated by miR1027, miR2619, miR5303, miR6114, and miR6455 [15]. Furthermore, it was found that three additional miRNAs regulate different types of transporters in watermelon—miR2662 for sugar transporters, miR5998 for sulfate and sugar transporters, and miR7517 for sucrose transporters. Additionally, one of the novel miRNAs identified by Liu et al. [17], miRn8-3p, acts as a nitrate transporter, which is associated with crop yields [16,17]. Only one miRNA has been found to regulate transport during CMV infection—miR408, which regulates copper transport in tomato [123]. Currently, no studies have demonstrated any effect on transport in cucurbit plants.

miRNAs have been shown to play an extensive role in regulating metabolism during viral infection. For cucurbits, this effect is best understood in watermelon, where miRNAs regulate the phenylpropanoid biosynthetic pathway and the fatty acid signaling pathway [15]. The phenylpropanoid biosynthetic pathway, responsible for the synthesis of lignin, flavonoids, and anthocyanins, contains genes regulated by several miRNAs: miR156, miR838, miR858, miR894, miR1435, miR5257, miR5827, and miR5998. The fatty acid signaling pathway is regulated by miR894, miR5303, miR8051, and miR8578, all of which target enzymes involved in this pathway. Furthermore, the same study identified miR5303 as a regulator of cellulose synthesis.

In the case of cucumber, three novel miRNAs, identified by Liu et al. [17], were shown to regulate various metabolic processes [17]. csa-miRn1-3p, which targets *MYB* genes and UDP-glycosyltransferase, is involved in regulation of secondary metabolism. csa-miRn3-3p and csa-miRn5-5p, regulators of cell wall metabolism, participate in inducting changes in the cell wall, with csa-miRn5-5p being responsible for the cell wall degradation process. The most significant of these novel miRNAs is csa-miRn6-3p, which targets several genes responsible for lipid biosynthesis and metabolism, as well as acetyltransferase activity and zinc ion binding.

The effect of CMV on metabolic pathways has also been observed in other crop species. It is worth noting that miR159 is associated with the regulation of metabolism in tobacco, a process mediated by *MYB* and *bZIP* transcription factors [127]. In tomato, miR390 has been linked to chlorophyll and heme biosynthesis, while miR395 targets an ATP sulfurylase, which is responsible for sulfate assimilation and the synthesis of sulfur-containing molecules [123,128]. Based on this, in can be concluded that miRNAs influence metabolic processes during CGMMV and CMV infection. However, this data remains less understood in CMV compared to CGMMV.

#### 4.5.4. Plant Developmental Processes

The changes in plant development result from the actions of miRNAs at several regulatory levels, some of which have been mentioned in the previous sections. In addition, certain miRNAs may also specifically regulate plant growth processes. All of these regulatory mechanisms contribute to the response to viral infection, which can be observed through changes in the plant’s appearance.

One of the best-known processes regulated by miRNAs in plant development is the regulation of flowering time. The key miRNA families involved in this regulation, miR156 and miR171/miR172, have been identified as fulfilling such a role during CMV infection in tomato [122,123]. The same study demonstrated the role of miR171 and miR172 in flower organ differentiation [122,123], while miR156 was assigned a role in regulation of flowering also in cucumber, where it cooperates with miR2673, miR2936 and miR3638, along with an additional role in pollen maturation [17]. Flower development has also been linked to miRNAs such as miR159 in tomato, acting as a regulator of floral transitions, flowering time and flower initiation during CMV infection [122,128], miR167 in flower maturation in tomato [122,123,128] as well as miR390 and the novel miRNA csa-miRn2-3p in cucumber [16,17]. Furthermore, miR159 and miR390 were also responsible for regulating fruit development in the case of CMV and CGMMV, respectively [17,122,128].

A number of miRNAs have been linked to the regulation of cellular processes. For instance, miR408, miR1027, miR2873, miR5303, and miR8578 are responsible for cell wall modulation in watermelon, which may mitigate the effects of viral infections [15]. Similarly, novel miRNAs csa-miRn1-3p, csa-miRn3-3p, and csa-miRn5-5p have been linked to cell morphogenesis and differentiation in cucumber [16,17]. It has also been shown that miR169 regulates embryo morphogenesis and cell differentiation in tobacco, as well as cytoskeletal organization in tomato [123,127].

Meristem development has been shown to be regulated during CMV infection by miR164, miR165, miR166 and miR319, which are responsible for proper differentiation, development and senescence of leaves, and additionally by miR160 in root cap development [123,126,127,128,129,130,131]. Meristem initiation in CGMMV-infected cucumber has been linked to the novel miRNA csa-miRn1-3p, whereas csa-miRn6-3p has been found to regulate both growth and senescence [16,17]. Regulation of miR396, which targets growth factors from the *GRF* family, causes leaf malformation, plant dwarfism, and yield losses [17,134,135].

These results demonstrate the extent to which miRNAs regulate plant growth, highlighting the importance of specific, conserved miRNA families for maintaining key plant developmental processes, and providing examples of novel miRNAs that regulate species-specific processes. The miRNAs presented respond to viral infection in a manner that induces structural changes in the infected plant during the viral attack.

#### 4.5.5. Interactions with lncRNAs and circRNAs

Another area of interest regarding miRNA activity is their interaction with other non-coding RNA molecules, notably lncRNAs and circRNAs. Analysis of watermelon’s response to CGMMV identified many lncRNAs and circRNAs interacting with miRNAs downstream, which in turn regulated their own target genes, forming a multi-layered pathway. Of these miRNAs, 13 of them (miR156, miR159, miR167, miR168, miR172, miR396, miR482, miR4995, miR5368, miR6300, miR9410, miR11610 and a novel miRNA) were assigned to the lncRNA/circRNA-miRNA-mRNA responding to the presence of CGMMV. The presence of both conserved and specific miRNAs underscores the significance of those pathways in plant–virus interactions.

### 4.6. Species-Specific and Virus-Specific miRNA Responses

Many miRNA families are evolutionally conserved, meaning that their functions remain consistent across diverse, distantly related species, and findings from well-studied species can be used to extrapolate putative functions of those miRNAs in newly studied plants. Nevertheless, the overall regulatory response to plant conditions is highly context-dependent and shaped by the adaptation of each species to its living conditions. Differences in miRNA expression profiles have been observed between cucumber and melon, demonstrating the diversity of miRNA regulation within the Cucurbitaceae family. Furthermore, responses to plant viruses such as CGMMV and CMV also appear to be regulated by specific miRNA interactions [15,16].

Several studies on miRNAs have shown that infections caused by CGMMV and CMV induce viral responses and symptoms that—although often regulated by the same miRNAs—differ in their specific mode of response, leading to distinct outcomes (Table 2). This suggests that specific viruses elicit specific regulatory responses in the host. At the same time, miRNA responses to viral infections were similar regardless of specific interspecies interactions, engaging in plant development, signal transduction, transport, metabolism and the molecular response to the infection, suggesting that they play similar roles between species. Given the paucity of data on miRNAs and their condition-specific detection, it remains to be determined exactly how differences in miRNA expression are shaped across species and viral diseases.

Among the analyzed studies, of particular note is the identification of novel miRNAs in cucumber [16,17,120] and several non-conserved miRNAs detected in watermelon [15], which play a role in the regulation of plant immunity and symptom development, often interacting with conserved miRNA families. These miRNAs may represent species-specific regulators involved in stress adaptation and mediating plant responses to pathogens. This highlights the importance of both conserved and species-specific miRNAs in inducing antiviral responses in cucurbits as part of a larger regulatory network.

Taken together, these studies indicate that although individual miRNA families regulate distinct biological processes, their coordinated activity forms an interconnected regulatory network integrating development, hormone signaling, RNA silencing, and antiviral defense.

### 4.7. Key Findings on miRNA-Mediated Anti-Viral Defense in Cucurbitaceae

Although numerous virus-responsive miRNAs have been identified across different experimental systems, several conserved regulatory patterns consistently emerge from the available studies. Among the numerous virus-responsive miRNAs identified to date, miR156, miR159, miR168, miR169, miR171, and miR172 emerge as the most consistently reported regulators across cucurbit-virus interactions, which are showcased through multiple studies (Table 2). miR168 regulates biosynthesis of all miRNAs, having an essential role in mediating the RNA silencing response against CMV [119], and also playing a role in the stress response against CGMMV [120]. miR159 is heavily involved in mediation of plant–virus interactions, causing induction of viral symptoms, as well as the defense response to the virus [16,120,121]. miR169 affects disease resistance through regulation of signal transduction, which may be associated with hormone defense pathways, and also influences the expression of resistance genes [16,17,121,123]. miR156, miR171 and miR172 are all important regulators of plant growth, which can be affected by viral infections, leading to developmental changes [15,16,17,120,121,132]. Those miRNA families constitute central regulatory hubs connecting developmental pathways, hormone signaling, RNA silencing, and disease resistance mechanisms (Figure 1). Their recurrent identification across independent studies suggests that they represent the core miRNA-mediated antiviral regulatory framework currently known in Cucurbitaceae.

Based on collected findings, several other miRNAs have also been presented as factors responsible for the miRNA regulation and influencing plant resistance. These miRNAs may perform a complimentary, condition- and species-specific role in the regulation of plant processes during viral infection, allowing the plant to adapt to environmental conditions and evolutionary pressure from their viral threats. Studies made on Cucurbitaceae plants showcase that the plant response to specific viruses, such as CMV and CGMMV, is a result of influence mediated by many miRNAs, including both conserved and more specific molecules [15,16,17,19,127,128,139,141]. This review highlights miR160, miR162, miR164, miR165/166, miR167, miR319, miR390, miR393, miR396, miR403, miR408, miR482, miR838 as other notable miRNAs contributing to regulatory networks mediated by the key regulatory miRNAs (Figure 2). This showcases the complex, multilayered nature of the miRNA interactions during viral response.

Furthermore, a few of the miRNAs studied (miR156, miR159, miR164, miR165/166, miR172, miR319, miR390, and miR396) have been identified as responsible for induction of disease symptoms (Figure 2). The viral influence on the expression of those miRNAs may alter developmental processes regulated by them, causing visual and structural changes that may lead to crop yield and quality losses [17,118,122,123,125,126,127,128,129,130,131,134,135]. These results show that miRNA expression is a significant factor affecting plant health. Further studies of this dynamic may therefore provide important knowledge on how to mitigate the harmful effect of crop-infecting viruses.

## 5. Experimental Validation of miRNA–Target Interactions in *Cucurbitaceae*

Predictions of miRNA–target interactions based on bioinformatic analyses require experimental validation to confirm their biological relevance [144]. In Cucurbitaceae species, including cucumber (*Cucumis sativus*), melon (*Cucumis melo*), watermelon (*Citrullus lanatus*), and pumpkin (*Cucurbita* spp.), several complementary approaches have been employed to verify miRNA-guided regulation [145,146,147,148]. These methods range from the identification of miRNA-mediated cleavage sites using degradome sequencing and 5’ RLM-RACE, which provide direct evidence of miRNA-guided transcript cleavage, to transient expression assays enabling rapid verification of target recognition [11,148,149]. Functional approaches, including virus-induced gene silencing (VIGS), overexpression studies, and genome editing, further elucidate the biological significance of miRNA-mediated regulation in plant growth, development, stress responses and antiviral defense [146,148]. Together, these complementary methods provide a robust framework for understanding miRNA function in Cucurbitaceae crops. However, the level of experimental evidence supporting individual miRNA–target interactions varies considerably among published studies. Therefore, distinguishing between computational predictions, expression-supported interactions, and experimentally validated targets is essential for accurately interpreting the current state of knowledge. Future studies integrating these experimental approaches with transcriptomics, small RNA sequencing, degradome analyses, and other multi-omics datasets will be crucial for establishing casual regulatory networks underlying antiviral defense in Cucurbitaceae.

### 5.1. Degradome Sequencing

Degradome sequencing, also known as Parallel Analysis of RNA Ends (PARE), is a high-throughput approach widely used to identify transcripts cleaved by miRNAs [149]. The method captures and sequences uncapped polyadenylated RNA fragments generated after AGO-mediated cleavage, enabling genome-wide detection of miRNA targets [149].

In *Cucurbitaceae*, degradome analyses have been extensively applied to validate computionally predicted targets of conserved and species-specific miRNAs in cucumber, watermelon, and other cucurbit crops [146,147,148]. This technique provides direct evidence of cleavage events and allows classification of targets according to the abundance of degradome signatures. Because plant miRNAs typically regulate gene expression through transcript cleavage, degradome sequencing has become one of the most reliable approaches for large-scale validation of miRNA–target interactions [11,150].

### 5.2. 5′RNA Ligase-Mediated Rapid Amplification of cDNA Ends (5′ RLM-RACE)

The 5′ RLM-RACE technique is commonly used to validate individual miRNA-directed cleavage sites identified by degradome sequencing or bioinformatic prediction [144]. The method involves ligation of an RNA adaptor to the 5′ ends of cleaved transcripts, followed by reverse transcription, PCR amplification, and sequencing of the resulting products.

This approach enables precise mapping of cleavage sites, which are typically located between the tenth and eleventh nucleotides complementary to the miRNA sequence [144]. In Cucurbitaceae studies, 5′ RLM-RACE has frequently been employed to confirm the regulation of transcription factors and stress-responsive genes targeted by specific miRNAs [148].

### 5.3. Transient Expression Assays

Transient expression systems provide a rapid method for assessing miRNA–target interactions in vivo. Typically, a reporter gene such as GFP, YFP, or luciferase is fused to the target sequence and co-expressed with the corresponding miRNA construct in plant tissues, often through Agrobacterium-mediated infiltration.

A reduction in reporter activity indicates effective miRNA-mediated regulation, whereas mutation of the miRNA-binding site can abolish this effect and serve as an important control [151]. Transient assays allow direct examination of target recognition and regulatory efficiency without the need to generate stable transgenic plants. In Cucurbitaceae research, transient expression analyses have been used to validate candidate miRNA targets and investigate the strength and specificity of miRNA-mediated regulation [152].

### 5.4. Virus-Induced Gene Silencing (VIGS)

Virus-induced gene silencing represents a valuable reverse-genetics tool for investigating the biological functions of miRNAs and their target genes. VIGS exploits modified viral vectors to induce sequence-specific silencing in plant tissues [153].

In *Cucurbitaceae* species, VIGS has been widely adopted because stable genetic transformation remains challenging in several economically important cultivars [154]. Silencing of target genes or components of the miRNA biogenesis pathway allows researchers to assess phenotypic consequences and establish functional links between miRNAs and developmental or stress-response processes [155].

### 5.5. Overexpression Approaches

Overexpression of miRNA precursors or target genes is commonly used to investigate the functional significance of miRNA-mediated regulation [151]. Transgenic plants expressing elevated levels of a specific miRNA frequently exhibit reduced accumulation of target transcripts and characteristic developmental or physiological phenotypes.

Conversely, overexpression of miRNA-resistant target genes carrying mutations in the miRNA-binding site can help demonstrate that observed phenotypes result directly from miRNA–target interactions [156]. In Cucurbitaceae, overexpression and functional studies have contributed substantially to understanding the roles of miRNAs in fruit development, flowering regulation, and environmental responses [148,156,157].

### 5.6. Genome Editing Technologies

Recent advances in genome editing, particularly CRISPR/Cas systems, have provided powerful tools for functional validation of miRNA–target interactions [158]. Genome editing can be used to disrupt miRNA genes, modify target loci, or precisely alter miRNA-binding sites without affecting protein-coding sequences [156].

Mutation introduced into target recognition sites can eliminate miRNA-mediated regulation and reveal the biological importance of individual interactions [158]. Similarly, knockout of miRNA genes allows direct assessment of their contribution to plant development and environmental responses.

## 6. Regulatory Networks and Cross-Talk

The antiviral activity of plant miRNAs is increasingly understood within the context of complex regulatory networks. In virus-infected plants, miRNAs operate as central nodes connecting RNA silencing, transcriptional regulation, hormone signaling, and broader non-coding RNA systems (Figure 2). In Cucurbitaceae, high-throughput sequencing studies have demonstrated that viral infection induces coordinated reprogramming of miRNA expression alongside widespread transcriptional changes, suggesting a system-level regulatory response rather than isolated gene modulation [15,16,17]. Collectively, these observations support the view that antiviral defense in cucurbits emerges from dynamic interactions among interconnected regulatory modules rather than from the activity of single resistance-associated genes.

### 6.1. miRNA–mRNA Interaction Networks as Regulatory Hubs

At the core of these responses are miRNA–mRNA interaction networks, in which individual miRNAs regulate multiple targets while individual genes are often controlled by several miRNAs. In cucurbits, virus-responsive miRNAs target mainly transcription factors, linking miRNA activity to key developmental and defense pathways [16,17]. These transcription factors act as regulatory hubs, amplifying miRNA effects across multiple downstream processes.

Beyond their direct antiviral roles, miRNA-regulated transcription factors contribute to large-scale transcriptional reprogramming during infection. *SPL* family members controlled by miR156 participate in developmental timing, phase transition, and hormone-responsive defense regulation [159], whereas *MYB* transcription factors targeted by miR159 are associated with SA and JA dependent signaling pathways as well as ABA-responsive stress regulation [160]. Similarly, *bZIP* and *NAC* transcription factors are closely linked to stress-responsive transcriptional cascades, systemic acquired resistance (SAR), and abiotic stress adaptation. Through simultaneous regulation of multiple transcription factors, miRNAs provide both robustness and plasticity to antiviral responses, enabling infected plants to rapidly balance growth-related and defense-related programs under changing physiological conditions.

The miR168–AGO1 regulatory module is another prominent example of miRNA–target interplay, acting as a crucial regulator of the plant’s entire defense system during infection. miR168 directly targets AGO1, thereby controlling the core component of the RNA silencing machinery. During viral infection, miR168 levels are frequently altered, resulting in changes in AGO1 accumulation, allowing AGO1 to form RISC, bind to vsiRNAs, and stabilize miR168. This regulatory loop represents a critical control point that can be exploited by viral suppressors, particularly CMV 2b, to fine-tune host silencing capacity by interfering with AGO1 activity, attenuating the antiviral response [18,20,161,162,163].

### 6.2. Cross-Talk with Hormone Signaling and Stress Pathways

miRNA-mediated regulatory networks are tightly integrated with plant hormone signaling pathways, which coordinate growth, development, and defense. Viral infection frequently alters pathways associated with SA, JA, auxins, and cytokinins, and miRNAs serve as important mediators of these changes [12,164].

For example, miR159 is associated with *MYB* transcription factors involved in hormone signaling, while miR393—widely studied in other plant systems—regulates auxin receptors and contributes to immune responses [165,166]. In cucurbits, predicted targets of virus-responsive miRNAs include genes involved in cytokinin metabolism and auxin signaling, suggesting that miRNA-mediated regulation contributes to balancing defense responses with developmental processes [16,167,168]. This hormonal cross-talk is particularly important because many disease symptoms reflect disruptions in growth-defense balance rather that direct viral cytotoxicity.

Viral infection also activates overlapping biotic and abiotic stress pathways, including oxidative stress, calcium signaling, mitogen-activated protein kinase (MAPK) cascades, and secondary metabolite biosynthesis. Increasing evidence suggests that miRNAs are deeply integrated into these stress-responsive networks, acting as intermediates between antiviral immunity and broader stress adaptation mechanisms. Several differentially expressed miRNAs identified in infected cucurbits are predicted to target genes associated with reactive oxygen species (ROS) detoxification, membrane transport, heat-shock proteins, and redox homeostasis [16,17]. Because ROS accumulation in a hallmark of antiviral defense, miRNA-dependent regulation of antioxidant systems may critically influence the balance between effective viral restriction and oxidative damage to host tissues.

Furthermore, stress-induced signaling pathways frequently converge on hormone-responsive transcription factors, creating multilayered feedback loops between miRNAs, hormonal regulation, and immune responses. Such integration likely contributes to symptom severity, tissue recovery, and systemic signaling during chronic viral infections. Emerging evidence also indicates interactions between RNA silencing pathways and autophagy-related processes, suggesting that miRNAs may indirectly influence selective degradation of viral components and damaged cellular structures during infection.

### 6.3. Integration with Other Non-Coding RNAs

Recent studies have expanded the regulatory landscape by demonstrating that miRNAs interact with other classes of non-coding RNAs, including lncRNAs and circRNAs, through ceRNA mechanisms. These molecules can modulate miRNA availability by sequestering them and thereby influencing target gene expression [120,169].

According to the ceRNA model, lncRNAs and circRNAs compete with mRNAs for miRNA binding, creating additional layers of post-transcriptional regulation during viral infection. Through this mechanism, non-coding RNAs can indirectly regulate defense-associated genes by altering the abundance of free, functional miRNAs within the cell. This expands the antiviral regulatory network beyond canonical miRNA–mRNA interactions and introduces a highly dynamic layer of RNA-based regulatory control [170].

In cucurbit species, integrative transcriptomic analyses have identified lncRNA–miRNA–mRNA and circRNA–miRNA interaction networks responsive to CGMMV infection, indicating that these additional RNA layers contribute to antiviral responses [120]. Several differentially expressed circRNAs were predicted to function as miRNA sponges associated with defense-related pathways, transcriptional regulation, and stress-responsive signaling. Although functional validation remains limited, these findings suggest that miRNA activity is embedded within multilayered regulatory systems that extend beyond classical RNA silencing pathways. Similar ceRNA architectures have also been described in other plant-virus systems, supporting the hypothesis that integrated non-coding RNA networks represent a conserved feature of antiviral immunity in plants.

### 6.4. Network Reprogramming During Viral Infection

Viral infection induces large-scale reorganization of host regulatory networks. Changes in miRNA expression can propagate through transcriptional and signaling cascades, affecting diverse biological processes including cell wall modification, transport, metabolism, and stress responses [15,16,17,168]. Consequently, antiviral responses should be viewed as coordinated systems-level adaptations involving extensive network rewiring.

Moreover, viral suppressors of RNA silencing, such as CMV 2b and potyviral HC-Pro, disrupt AGO-mediated pathways and interfere with miRNA function, leading to system-wide alterations in gene expression [18,20,162]. Beyond suppressing antiviral silencing directly, these viral proteins may perturb broader regulatory circuits controlling hormone signaling, stress responses, and developmental pathways. Such interference can disrupt pattern-triggered immunity, alter defense-associated transcriptional programs, and reshape small RNA accumulation profiles throughout infected tissues.

These disruptions contribute not only to viral replication but also to symptom development, reinforcing the idea that disease phenotypes arise from network-level perturbations rather than solely from direct viral damage. Symptoms such as chlorosis, mosaic patterns, leaf deformation, and growth inhibition likely reflect widespread deregulation of interconnected RNA-guided signaling pathways and loss of regulatory homeostasis.

### 6.5. Implications for Systems Biology and Crop Protection

Understanding miRNA-mediated antiviral responses from a network perspective has important implications for crop protection. Rather than targeting individual genes, future strategies may focus on key regulatory nodes within these networks, such as miRNA hubs, AGO-centered modules, or hormone-linked regulatory circuits. Such systems-oriented approaches may provide more durable resistance by targeting central regulatory architectures that are more difficult for viruses to overcome evolutionarily.

Emerging technologies such as degradome sequencing, single-cell transcriptomics, spatial transcriptomics, and network-based computational modeling are expected to substantially improve understanding of miRNA-centered antiviral regulation in cucurbits. Integration of transcriptomic, small RNA, degradome, and epigenomic datasets may help identify master regulatory nodes with disproportionate influence on infection outcomes and symptom development. From an applied perspective, engineering miRNA regulatory pathways represent a promising avenue for developing virus-resistant cucurbit crops. Potential strategies include artificial miRNAs (amiRNAs), CRISPR/Cas-mediated editing of miRNA target sites, manipulation of AGO-associated pathways, and synthetic target mimics or decoy RNAs designed to modulate miRNA availability [16,171]. However, a major limitation in current research is the lack of functional validation of predicted interactions. While high-throughput data have revealed complex regulatory architectures, experimental studies confirming these networks remain scarce.

Addressing this gap will require integrated multi-omics approaches combining transcriptomics, small RNA sequencing, and functional assays. Nonetheless, miRNA-centered regulatory networks represent a promising framework for understanding plant–virus interactions and developing next-generation RNA-based strategies for improving resistance in cucurbit crops.

## 7. Dual Role of MicroRNAs in Virus Infection

Plant miRNAs play a dual and often paradoxical role during viral infection. On one hand, they function as essential components of antiviral defense by regulating immune signaling, RNA silencing pathways, and stress-responsive gene expression. On the other hand, virus-induced alterations in miRNA accumulation can disrupt developmental and physiological processes, contributing directly to symptom formation and disease progression. In Cucurbitaceae, increasing evidence suggests that many disease phenotypes arise not solely from viral replication itself, but also from extensive miRNA-mediated reprogramming of host regulatory networks [15,17,20].

Importantly, miRNA-mediated defense responses are highly dynamic and context-dependent. Depending on the virus species, infection stage, tissue specificity, and environmental conditions, the same miRNA may exhibit either protective or susceptibility-associated functions [7,14]. This functional plasticity highlights the complexity of miRNA-centered antiviral regulation in plants and suggests that miRNAs should be viewed both as defense regulators and as potential drivers of pathogenic symptom development.

### 7.1. miRNAs as Regulators of Antiviral Defense

During infection, host plants rapidly alter the expression of numerous conserved and species-specific miRNAs involved in immune regulation. These changes contribute to antiviral defense by modulating RNA silencing components, hormone signaling pathways, stress-responsive transcription factors, and genes associated with systemic immunity [12,14]. In cucurbits, virus-responsive miRNAs such as miR156, miR159, miR168, miR171, and miR398 have been associated with defense-related transcriptional reprogramming during infections caused by CMV, ZYMV, and CGMMV [15,16,17].

One of the best-characterized examples of antiviral regulation is the miR168–AGO1 regulatory module. AGO1 is a central component of RISCs, and its expression is tightly controlled by miR168. Viral suppressors of RNA silencing, such as the CMV 2b protein and potyviral HC-Pro, interfere with AGO1-associated pathways, thereby manipulating endogenous miRNA regulation to weaken host antiviral defenses and enable viral replication [18,20]. Other miRNAs contribute indirectly to defense by regulating phytohormone signaling and stress adaptation. For instance, miR159 regulates *MYB* transcription factors associated with SA, JA, and abscisic acid (ABA) signaling pathways [12,160], while miR156-controlled *SPL* transcription factors participate in developmental timing and defense-related regulation [172]. Such regulatory interactions allow plants to coordinate immune activation with growth maintenance during infection.

### 7.2. miRNAs as Drivers of Disease Symptoms

Although miRNAs contribute to antiviral defense, dysregulation of miRNA pathways during infection can also promote symptom development. Viral suppressors of RNA silencing frequently disrupt endogenous miRNA homeostasis, leading to abnormal expression of developmental regulators and large-scale transcriptional imbalance [14,20]. Consequently, many disease symptoms observed in infected cucurbits may result from altered host gene regulation rather than direct cytopathic effects caused by viral replication.

Leaf deformation, mosaic symptoms, chlorosis, and abnormal organ development are commonly associated with perturbations in miRNA-regulated pathways. In particular, virus-induced changes in miR164, miR165/166, and miR319 expression have been linked to altered leaf polarity, impaired cell differentiation, and defects in tissue morphogenesis in several plant species [165,173,174]. Because these miRNAs regulate *NAC*, *HD-ZIP*, and *TCP* transcription factors involved in organ development, disruption of their expression can profoundly affect leaf architecture and vascular organization. Similar mechanisms are likely involved in cucurbit viral pathogenesis, where severe leaf curling, blistering, and mosaic phenotypes are frequently observed during CMV and potyvirus infections [15,17].

Viral infection can also interfere with developmental timing and reproductive transitions through miRNA-mediated pathways. The miR156–*SPL* regulatory module, which controls phase transition and flowering time, is particularly important in this context [175]. Elevated miR156 accumulation during stress conditions suppresses *SPL* transcription factors and can delay flowering and developmental progression [175,176]. In virus-infected plants, dysregulation of this pathway may contribute to delayed flowering, reduced reproductive fitness, and impaired fruit production. Such developmental alterations are especially important in cucurbit crops because viral diseases often significantly reduce yield through combined effects on vegetative growth and reproductive development.

Additionally, perturbation of hormone-associated miRNAs may contribute to symptom severity by altering the balance between growth and defense signaling. Changes in auxin-, cytokinin-, and gibberellin-related pathways frequently correlate with stunted growth, reduced internode elongation, and abnormal fruit development in virus-infected plants [12,165]. Thus, symptom formation should be considered an emergent consequence of extensive network-level disruption involving miRNAs, hormone signaling, and transcriptional regulation.

### 7.3. Viral Manipulation of Host miRNA Pathways

Many plant viruses actively manipulate host miRNA pathways to facilitate infection and systemic spread. VSRs not only inhibit antiviral siRNA activity but also interfere with endogenous miRNA biogenesis, AGO loading, and small RNA stability [20,177]. These interactions may alter gene expression patterns associated with immunity, development, and metabolism.

For example, HC-Pro proteins encoded by potyviruses can bind small RNAs and interfere with their incorporation into AGO complexes, whereas CMV 2b directly interacts with AGO proteins and suppresses RNA silencing activity [18]. Because endogenous miRNAs share components of the silencing machinery with antiviral siRNAs, viral interference often leads to widespread deregulation of host developmental programs. As a result, symptom development may reflect the collateral consequences of viral suppression of small RNA homeostasis.

In some cases, viruses may selectively exploit host miRNAs to enhance susceptibility. Virus-induced upregulation of specific miRNAs can suppress defense-associated genes, creating favorable conditions for viral accumulation, whereas downregulation of protective miRNAs may impair stress adaptation and immune signaling [7,14]. These observations reinforce the concept that plant viruses function both as pathogens and as sophisticated modulators of host regulatory networks.

## 8. RNA-Based Strategies for Enhancing Virus Resistance in Cucurbitaceae

The growing understanding of RNA silencing and miRNA-mediated regulatory networks has opened new avenues for developing virus-resistant crops. In Cucurbitaceae, where viral diseases remain a major constraint on productivity, RNA-based approaches are increasingly considered promising alternatives to conventional breeding and chemical control strategies. These approaches exploit endogenous gene regulation mechanisms and offer potential for high specificity and environmental safety.

### 8.1. Artificial MicroRNAs and Targeted Gene Silencing

amiRNAs represent one of the most precise tools for virus resistance engineering. Designed to mimic endogenous miRNAs, amiRNAs can be tailored to target viral genomes or essential viral genes, thereby reducing viral replication and systemic movement. In model plants, Niu et al. [178] demonstrated that amiRNAs based on the *Arabidopsis* miR159 precursor could target viral suppressor genes, including P69 of turnip yellow mosaic virus and HC-Pro of turnip mosaic virus. Transgenic *Arabidopsis* plants expressing amiR-P69 or amiR-HC-Pro showed resistance to the corresponding viruses, while plants expressing a dimeric pre-amiRNA construct showed resistance to both viruses [178].

In Cucurbitaceae, Berbati et al. [179] designed four amiRNAs based on the *Arabidopsis* miR390a backbone to target the HC-Pro region of ZYMV. Agroinfiltration of zucchini cotyledons with these amiRNAs reduced viral titers and delayed symptom development, resulting in 16–42.5% protection at 21 days post-inoculation compared with the control amiRNA targeting tobacco mosaic virus [179].

In cucumber, polycistronic amiRNA constructs have been developed to target conserved regions of CGMMV. Miao et al. [171] designed amiRNAs against CGMMV genomic regions encoding replication-associated proteins, movement protein, and coat protein. Stable transgenic cucumber plants expressing polycistronic amiRNA showed enhanced resistance to CGMMV, and no sequence mutation was detected in CGMMV, indicating that this strategy may reduce the risk of viral escape [171].

### 8.2. RNA Interference (RNAi) and Transgenic Approaches

RNA interference has long been recognized as a powerful antiviral strategy in plants. Transgenic expression of double-stranded RNA corresponding to viral genes can trigger the production of virus-derived siRNAs, leading to sequence-specific degradation of viral RNA. In Cucurbitaceae, transgenic cucumber and melon lines carrying a hairpin construct of the ZYMV HC-Pro gene displayed resistance to systemic ZYMV infection [180]. One cucumber line with elevated levels of transgene-derived siRNAs and increased expression of RDR1 and AGO1 also showed resistance to watermelon mosaic virus and papaya ringspot virus-W, suggesting that strong transgenic small-RNA accumulation may contribute to broader potyvirus resistance [180].

Host-factor silencing has also been tested in melon. Rodríguez-Hernández et al. [181] generated melon RNAi lines with reduced expression of *Cm-eIF4E*, a host susceptibility factor required by several viruses. These T2 transgenic melon plants were resistant to four of eight tested melon-infecting viruses: ZYMV, Moroccan watermelon mosaic virus, cucumber vein yellowing virus, and melon necrotic spot virus [181]. However, transgenic approaches face regulatory and public acceptance challenges in many regions. These limitations have encouraged the development of non-transgenic RNA-based strategies.

### 8.3. Exogenous dsRNA and Non-Transgenic Approaches

Recent advances have led to the development of non-transgenic RNA-based technologies, including the exogenous application of dsRNA molecules to plants. This approach, often referred to as spray-induced gene silencing, can induce RNAi without stable genetic modification. In Cucurbitaceae, Kaldis et al. [182] showed that exogenously applied dsRNAs derived from the HC-Pro and CP genes of ZYMV protected cucumber, watermelon, and squash against ZYMV. HC-Pro-derived dsRNA conferred resistance of 82%, 50%, and 18% in cucumber, watermelon, and squash, respectively, while CP-derived dsRNA conferred resistance of 70%, 43%, and 16%, respectively [182].

RT-PCR analyses confirmed that exogenously applied dsRNA molecules were detected in systemic tissues of watermelon and squash and remained detectable for at least nine days after application. The detection of ZYMV-derived siRNAs indicated that these dsRNAs were processed by the plant RNA silencing machinery [182].

This strategy offers flexibility, reduced regulatory barriers relative to transgenic approaches, and potential rapid adaptation to emerging viral strains. Nevertheless, major challenges remain, especially dsRNA stability, delivery efficiency, uptake by plant tissues, formulation, and field performance [183]. Recent work in cucurbits has also shown that exogenous dsRNA can reduce symptoms of CGMMV in cucumber and ToLCNDV in zucchini, further supporting the relevance of this approach for Cucurbitaceae [184].

### 8.4. Cross-Kingdom Activity of Plant miRNAs

Recent studies have demonstrated that plant-derived miRNAs may function beyond species boundaries and participate in cross-kingdom regulatory interactions. Plant miRNAs have been shown to influence gene expression in fungi, bacteria, and animal systems following their transfer through extracellular vesicles or other transport mechanisms such as transformation or transfection [185,186,187]. Such observations indicate that plant miRNAs can act as mobile signaling molecules capable of modulating biological processes in interacting organisms. Although direct evidence linking cross-kingdom regulation to antiviral defense in Cucurbitaceae remains limited, these findings highlight the remarkable regulatory versatility of plant miRNAs and support the view that they may participate in broader host–pathogen communication networks. Cross-kingdom RNA communication may therefore provide new perspectives for developing RNA-based crop protection strategies. However, direct evidence for cross-kingdom miRNA-mediated antiviral defense in Cucurbitaceae remains limited, and this concept should currently be regarded as an emerging research direction rather than an established mechanism.

## 9. Research Limitations and Future Perspectives

Despite substantial progress in understanding miRNA-mediated antiviral responses, several limitations continue to constrain both mechanistic interpretation and practical application in Cucurbitaceae. Before RNA-based strategies can be widely implemented in cucurbit crops, challenges related to functional validation, regulatory network complexity, developmental trade-offs, and field-level performance must be addressed.

### 9.1. Limited Functional Validation of miRNA–Target Interactions

One of the major gaps in current research on miRNA-mediated antiviral responses in cucurbits is the discrepancy between large-scale expression studies and functional validation. High-throughput sequencing has identified virus-responsive miRNAs in cucumber infected with CGMMV; however, most evidence remains based on differential expression profiling and computational target prediction rather than direct experimental confirmation of miRNA–target interactions or antiviral phenotypes [16,17].

Although computational predictions and degradome analyses provide valuable insights into potential regulatory networks and useful candidate interactions, they are insufficient to establish causal relationships between specific miRNAs, their targets, and antiviral resistance phenotypes. Moreover, plant miRNAs regulate gene expression through both target mRNA cleavage and translational repression. While degradome sequencing is effective for identifying cleavage events, it cannot detect targets regulated predominantly at the translational level. Studies in *Arabidopsis* have demonstrated that miRNA-mediated translational inhibition can occur on the endoplasmic reticulum, highlighting an additional regulatory layer that may remain undetected in studies relying solely on transcript abundance or degradome data [188].

Despite the increasing number of virus-responsive miRNAs identified in cucurbit crops, relatively few predicted miRNA–target interactions have been experimentally validated. Direct functional evidence based on degradome sequencing, 5′ RLM-RACE, reporter-based assays, virus-induced gene silencing (VIGS), target mimicry, overexpression, knockdown, or genome-editing approaches remains limited (Table 2). In particular, short tandem target mimic (STTM) technology has emerged as a powerful tool for miRNA loss-of-function studies and has been successfully applied to investigate miRNA regulatory networks in several plant species, demonstrating its potential for validating antiviral miRNA functions and identifying candidate targets for crop improvement [189].

Future research should therefore combine small RNA sequencing, transcriptomics, degradome sequencing, and lncRNA/circRNA profiling with targeted functional approaches capable of assessing both mRNA cleavage and translational repression. Integrating these complementary methodologies will be essential for establishing causal miRNA–target relationships, identifying key regulatory modules involved in antiviral defense, and translating these findings into disease-resistance breeding strategies for cucurbit crops.

### 9.2. Insufficient Understanding of Regulatory Network Complexity

miRNAs operate within multilayered regulatory systems involving transcription factors, hormone signaling pathways, stress-response modules, and other non-coding RNAs. However, most available studies still focus on individual miRNAs or predicted targets rather than dynamic network-level interactions.

In Cucurbitaceae, the integration of miRNAs with lncRNAs and circRNAs remains poorly characterized. In watermelon infected with CGMMV, genome-wide analyses identified lncRNA–miRNA–mRNA and circRNA–miRNA interaction networks, but their functional relevance remains largely untested [120]. Similar lncRNA/circRNA-associated regulatory layers have been described in tomato infected with TYLCV, suggesting that such networks may represent a broader feature of plant–virus interactions [169]. Future studies should therefore combine small RNA sequencing, transcriptomics, degradome analysis, and functional assays to clarify how these regulatory layers interact during antiviral responses.

### 9.3. Lack of Field-Based and Translational Studies

Another major limitation is the predominance of laboratory-based studies conducted under controlled conditions. These experiments provide important mechanistic insights, but they do not fully capture the complexity of plant–virus interactions in agricultural environments.

Temperature, light, water availability, plant developmental stage, vector pressure, mixed infections, and concurrent abiotic stresses can influence RNA silencing and miRNA expression. The performance of RNA-based technologies, including RNAi, artificial miRNAs, and exogenous dsRNA, may therefore differ between laboratory and field conditions. In particular, exogenous RNA approaches still face challenges related to dsRNA stability, formulation, delivery, uptake, systemic movement, and persistence under field conditions [183,184].

### 9.4. Balancing Defense and Developmental Trade-Offs

A recurring challenge in miRNA-based crop improvement is the dual role of miRNAs in defense and development. Many miRNAs associated with antiviral responses also regulate flowering time, leaf morphology, phase transition, hormone signaling, and stress adaptation. This functional overlap creates potential trade-offs. Enhancing antiviral resistance through miRNA manipulation may unintentionally affect growth, development, fertility, fruit quality, or yield. For example, the miR156–*SPL* module regulates developmental timing and stress-related responses, illustrating the difficulty of modifying miRNA pathways without pleiotropic effects [159]. Future strategies should therefore prioritize tissue-specific, inducible, or pathogen-responsive approaches that minimize developmental penalties.

### 9.5. Future Directions: Toward Integrated and Precision Approaches

Future research should prioritize multi-omics integration, including transcriptomics, small RNA sequencing, degradome analysis, epigenomics, proteomics, and phenotypic data. Such integration will support the construction of more reliable regulatory networks and help identify miRNA hubs, target genes, and RNA-based modules with disproportionate influence on infection outcomes.

CRISPR/Cas-based genome editing offers opportunities for precise manipulation of susceptibility genes, miRNA genes, miRNA target sites, and regulatory elements. These tools may complement artificial miRNAs, RNAi, and spray-induced gene silencing in the development of targeted antiviral strategies [190,191,192]. However, their use in Cucurbitaceae will require careful assessment of off-target effects, developmental consequences, regulatory constraints, and field performance.

Finally, research should expand beyond cucumber and watermelon to include melon, squash, pumpkin, and other economically important cucurbits. Comparative studies across host species and viral pathogens may reveal conserved regulatory mechanisms as well as species-specific adaptations. This will be essential for translating molecular knowledge into practical virus-resistance strategies for Cucurbitaceae.

## 10. Conclusions

MicroRNA-mediated regulation is an important component of antiviral responses in plants, linking RNA silencing with broader gene regulatory networks. In Cucurbitaceae, studies on CGMMV-infected cucumber and watermelon indicate that viral infection can alter miRNA expression, predicted miRNA–target interactions, and non-coding RNA-associated regulatory networks.

Rather than acting as isolated regulators, miRNAs appear to function as components of broader regulatory systems involving transcription factors, hormone signaling, stress-response pathways, and other non-coding RNAs. These networks may contribute not only to antiviral defense but also to symptom development and growth–defense trade-offs. However, current knowledge in cucurbit crops remains largely descriptive, and many predicted miRNA–target and ceRNA interactions still require functional validation.

From an applied perspective, RNA-based technologies, including artificial miRNAs, RNA interference, and exogenous dsRNA, provide promising tools for improving virus resistance in cucurbit crops. Their integration with genome editing and systems biology may enable more precise and durable resistance strategies. Nevertheless, practical deployment will require stronger functional evidence, field-based validation, and assessment of agronomic performance.

In conclusion, miRNA-centered regulatory networks represent important research frontiers in plant virology and crop protection. Future advances in Cucurbitaceae antiviral research will require the integration of multi-omics approaches, functional validation, and emerging insights into cross-kingdom RNA communication to establish a mechanistic understanding of miRNA-mediated defense networks and translate these findings into field-relevant strategies for virus resistance.

## Figures and Tables

**Table 2:** Host miRNA families identified as responding to CGMMV and CMV, their predicted or validated target genes, and proposed biological functions. Evidence levels were classified as CP (computational prediction), EE (expression-based evidence based on expression profiling or co-expression analysis), and EV (experimentally validated interaction). Where annotation was provided, target genes are referred to by their gene name, while genomic locus ID was used for other genes.

Host miRNA Families	Virus	Plant Species	Potential/Known Targets	Biological Function	Putative Role	References
miR156	CGMMV	*C. sativus*	*SPL* genes, *Csa6M091970.2*, *Csa6M091970.1*, *Csa5M198140.1*, *Csa1M038340.1* (EE)	Regulation of morphogenesis, pollen maturation, flowering time, microbial defense, signal transduction	Causes fruit and leaf deformation, delayed blooming, reduced fruit production	[17]
		*C. lanatus*	Phenylpropanoid biosynthetic pathway genes, *Cla006266* (CP)	Phenylpropanoid synthesis (lignin, flavonoids, anthocyanins), stress response	Plant growth and development, antimicrobial activity, response to CGMMV mediated by circRNAs	[15,120]
	CMV	*C. sativus*	*NBS-LRR* genes	Downregulation of plant resistance genes in absence of infection	Disease resistance	[121]
		*S. lycopersicum*	*SPL* genes (EE)	Promotion of floral transitions, flowering time	Delayed flowering time	[122,123]
miR157	CMV	*C. sativus*	*NBS-LRR* genes (EE)	Downregulation of plant resistance genes in absence of infection	Disease resistance	[121]
miR158	CMV	*A. thaliana*	Unknown	Regulation of plant development	Plant development	[124]
miR159	CGMMV	*C. sativus*	Transcription factor *MYB29*-like, salicylic acid-binding protein	Abiotic stress tolerance and biotic stress resistance, mediates interactions between the plant and the virus	Disease resistance	[16]
		*C. lanatus*	*Cla011748*, *Cla017037* (CP)	Stress response	Response to CGMMV mediated by lncRNAs and circRNAs	[120]
	CMV	*A. thaliana*, *N. benthamiana*	*MYB33*, *MYB65* (EV)	Inhibition induces symptoms caused by severe strains	Causes stunting and plant deformation	[125,126]
		*C. sativus*	*NBS-LRR* genes (EE)	Downregulation of plant resistance genes in absence of infection	Disease resistance	[121]
		*N. tabacum*	*MYB* and *bZIP* transcription factors	Plant development, hormone signaling, metabolism, response to biotic and abiotic stresses	Stress response	[127]
		*S. lycopersicum*	*MYB* genes, *GAMyb-like1* transcription factor	Promotion of floral transitions, flowering time, initiation of flowers, closed bud, flower and fruit development, hormone signaling	Altered leaf, flower and fruit development	[122,128]
miR160	CGMMV	*C. sativus*	Heterotrimeric G-protein encoding genes	Signal transduction	Disease resistance	[17]
	CMV	*N. tabacum*, *S. lycopersicum*	*ARF10*, *ARF17* (EV)	Plant development, root cap development, auxin response, signal transduction	Production of seedless fruit in tomato, regulation of signal transduction	[122,123,127]
miR162	CGMMV	*C. lanatus*	*LRR* and *RGA* genes, defense-related proteins	Regulation of resistance genes	Plant resistance to pathogens	[15]
	CMV	*C. sativus*	*NBS-LRR* genes	Downregulation of plant resistance genes in absence of infection	Disease resistance	[121]
		*N. tabacum*, *S. lycopersicum*	*DCL1* (EV)	miRNA biogenesis pathway, RNA silencing	Increased miRNA levels during infection	[122,127,129]
miR164	CMV	*N. tabacum*, *S. lycopersicum*	*NAC1*, *CUC2* (EV)	Plant development, Meristem initiation and maintenance, axillary meristem differentiation, formation of boundaries between meristems and organ primordia, leaf morphology, control of lamina outgrowth and serrations	Reduction in leaflet numbers, leaflet fusions, suppressing serrations, virus infection defense mechanism	[127,128,129,130,131]
miR165/166	CGMMV	*C. lanatus*	Unknown	Unknown	Unknown	[15]
	CMV	*A. thaliana*, *N. benthamiana*, *N. tabacum*, *S. lycopersicum*	*HD-ZIP III*, homeodomain-leucine zipper protein Revoluta (EV)	Meristem initiation and maintenance, axillary meristem differentiation, leaf morphology, disruption of adaxial/abaxial and medial/lateral features of leaf development, inhibition induces symptoms caused by severe strains, hormone signaling	Causes stunting, leaf and flower deformation, growth of enations from the veins on the abaxial surface	[118,123,126,127,128,129,130]
miR167	CGMMV	*C. lanatus*	*Cla002216* (CP)	Stress response	Response to CGMMV mediated by circRNAs	[120]
	CMV	*N. tabacum*	*PP2C*	Signal transduction	Regulation of plant development	[127]
		*S. lycopersicum*	*ARF6*, *ARF8* (EV)	Flower maturation, fruit initiation, auxin signaling, leaf development	Regulation of leaf, flower and fruit growth	[122,123,128]
miR168	CGMMV	*C. lanatus*	*Cla012782*, *Cla015459* (CP)	Stress response	Response to CGMMV mediated by circRNAs	[120]
	CMV	*A. thaliana*, *N. tabacum*, *S. lycopersicum*	*AGO1* (EV)	miRNA biogenesis pathway, RNA silencing, plant defense mechanism	Increased miRNA levels during infection, suppression of antiviral RNA silencing	[119,122,123,124,127,128,129]
miR169	CGMMV	*C. sativus*	Heterotrimeric G-protein encoding genes, putative disease resistance protein RGA3- like	Signal transduction, triggers a defense system that restricts pathogen growth	Disease resistance	[16,17]
	CMV	*C. sativus*	*NBS-LRR* genes	Downregulation of plant resistance genes in absence of infection	Disease resistance	[121]
		*N. tabacum*	CCAAT-binding transcription factor (CBF-B/NF-YA) family protein (EV)	Embryo morphogenesis, cellular differentiation	Plant development, stress response	[127]
		*S. lycopersicum*	Nuclear transcription factor *Y*, TGACG-sequence-specific DNA-binding protein gene, *NPR1*-interactor protein gene, complex protein 1 subunit gamma gene (EE)	Transduction of salicylic acid signal, cross-talk between salicylate- and jasmonate-dependent defense pathways, folding and assembly of cytoskeleton proteins, recognition of specific pathogens and initiation of defense response through R genes	Disease resistance, plant immunity response	[123]
miR171	CGMMV	*C. sativus*	*Csa6M109640.1*, *Csa3M020600.1*, *AP1/2* (EE)	Transcription regulation, growth, phloem transport, and flower organ differentiation	Transport of virus particles within infected plants	[17,132]
	CMV	*C. annum*, *N. tabacum*, *S. lycopersicum*	*SCL* genes (EV)	Promotion of floral transitions, flowering time	Regulation of flowering time, radical patterning of shoots and roots	[122,127,133]
miR172	CGMMV	*C. sativus*	*Csa6M109640.1*, *Csa3M020600.1*, *AP1/2*, Ethylene-responsive transcription factor *RAP2-7-like*	Transcription regulation, growth, phloem transport, and flower organ differentiation	Transport of virus particles within infected plants, regulation of genes by stress factors and signal transduction pathways	[16,17,132]
		*C. lanatus*	*Cla005135*	Stress response	Response to CGMMV mediated by circRNAs	[120]
	CMV	*C. sativus*	*NBS-LRR* genes	Downregulation of plant resistance genes in absence of infection	Disease resistance	[121]
		*S. lycopersicum*	*AP2* (EV)	Flowering time, flower morphology	Defective phenotype in flower and fruit organs	[122,123]
		*A. thaliana*	Unknown	Regulation of plant development	Plant development	[124]
miR319	CGMMV	*C. sativus*	*TCP* genes (EV)	Affects leaf number, suppression of cell proliferation, regulation of miR396	Leaf malformation, plant dwarfism, yield losses	[17,134]
	CMV	*C. annum*, *S. lycopersicum*	*TCP3*, *TCP4*, *TCP10*, *TCP24* (EV)	Meristem initiation and maintenance, leaf morphogenesis and senescence	Altered leaf morphology, defense response	[123,129,130,133]
miR390	CGMMV	*C. sativus*	Unknown	Regulation of developmental timing	Delayed flowering, reduced fruit production	[17]
		*C. lanatus*	*LRR* and *RGA* genes, defense-related proteins	Regulation of resistance genes	Plant resistance to pathogens	[15]
	CMV	*C. sativus*	*NBS-LRR* genes	Downregulation of plant resistance genes in absence of infection	Disease resistance	[121]
		*S. lycopersicum*	*PP2C*, coproporphyrinogen-III oxidase gene (CP)	Signal transduction, chlorophyll biosynthetic pathway, heme biosynthetic pathway	Regulation of plant development, regulation of chlorophyll and heme level in *Arabidopsis*	[123,128]
miR393	CGMMV	*C. lanatus*	Pectinesterase, pectinesterase inhibitors (CP)	Auxin signal perception, Aux/IAA degradation, affects related gene expression, cell wall modulation, host–cell receptor recognition	Mediating plant resistance to microbial attack	[15]
	CMV	*A. thaliana*	Unknown	Regulation of plant development	Plant development	[124]
miR395	CMV	*S. lycopersicum*	ATP sulfurylase 1	Sulfate assimilation	Synthesis of sulfur containing amino acids, lipid and coenzyme	[123]
miR396	CGMMV	*C. sativus*	*GRF* genes (EV)	Petal growth and development in *Arabidopsis*	Leaf malformation, plant dwarfism, yield losses	[17,134,135]
		*C. lanatus*	*Cla005204*, *Cla005206*, *Cla009071*, *Cla009720*, *Cla011362*, *Cla014800*, *Cla015355* (CP)	Stress response	Response to CGMMV mediated by lncRNAs and circRNAs	[120]
	CMV	*C. sativus*	*NBS-LRR* genes	Downregulation of plant resistance genes in absence of infection	Disease resistance	[121]
		*S. lycopersicum*	*GRF* genes, DNA cytosine 5-methyltransferase gene	DNA modification in prokaryotes	Genome management, regulation of development	[123]
miR398	CMV	*S. lycopersicum*	Cu/Zn superoxide dismutase, glycerol-3- phosphate transporter 1-like gene (EV)	Unknown	Unknown	[123]
miR403	CMV	*A. thaliana*	*AGO2* (EV)	RNA silencing	Antiviral RNA silencing, regulated by *AGO1*	[119]
miR408	CGMMV	*C. sativus*	Heterotrimeric G-protein encoding genes	Signal transduction	Disease resistance	[17]
		*C. lanatus*	Laccase 3 (CP)	Cell wall modulation, lignification and thickening of the cell wall	Alleviating virus attacks	[15]
	CMV	*S. lycopersicum*	Copper-transporting ATPase PAA2	Copper transport	Regulation in response to copper	[123]
miR477	CGMMV	*C. lanatus*	DELLA protein genes (CP)	Gibberellic acid signaling, modulation of salicylic-acid- and jasmonic-acid-dependent defense mechanisms	Plant immunity response	[15]
miR482	CGMMV	*C. lanatus*	*Cla003082*, *Cla013340* (CP)	Stress response	Response to CGMMV mediated by lncRNAs and circRNAs	[120]
	CMV	*C. annum*, *S. lycopersicum*	Putative disease-resistance RPP13-like NB-LRR protein gene, *NBS*-coding resistance (R) gene	Recognition of specific pathogens and initiation of defense response through R genes	Plant immunity response, plant development, disease resistance	[123,133]
miR530	CGMMV	*C. lanatus*	Eukaryotic translation initiation factors	Viral RNA replication	Mediating plant resistance to microbial attack	[15]
miR838	CGMMV	*C. sativus*	MACPF domain-containing protein CAD1-like (EE)	Regulation of SA- mediated pathway of programmed cell death during hypersensitivity response	Plant immunity response	[16]
		*C. lanatus*	Auxin transporter, *LRR* and *RGA* genes, defense-related proteins, phenylpropanoid biosynthetic pathway genes (CP)	Asymmetrical distribution of auxins, regulation of resistance genes, phenylpropanoid synthesis (lignin, flavonoids, anthocyanins)	Response to abiotic stress, plant resistance to pathogens, plant growth and development, antimicrobial activity	[15]
miR854	CGMMV	*C. sativus*	*WRKY* transcription factor 21-like (EE)	Mediates plant-pathogen interactions	Defense response, disease resistance	[16]
miR858	CGMMV	*C. lanatus*	Phenylpropanoid biosynthetic pathway genes (CP)	Phenylpropanoid synthesis (lignin, flavonoids, anthocyanins)	Plant growth and development, abiotic stress response, antimicrobial activity	[15]
miR894	CGMMV	*C. lanatus*	Phenylpropanoid biosynthetic pathway genes, fatty acyl-CoA reductase (CP)	Phenylpropanoid synthesis (lignin, flavonoids, anthocyanins), fatty acid signaling pathway	Plant growth and development, abiotic stress response, antimicrobial activity, response to virus infection	[15]
miR1027	CGMMV	*C. lanatus*	ABC transporters, pectinesterase, pectinesterase inhibitors, ethylene-responsive factors, *LRR* and *RGA* genes, defense-related proteins (CP)	Intracellular transport, cell wall modulation, host–cell receptor recognition, ethylene signaling, regulation of resistance genes	Mediating plant resistance to microbial attack, defense response, plant resistance to pathogens	[15]
miR1028	CGMMV	*C. lanatus*	Ethylene-responsive factors (CP)	Ethylene signaling	Defense response	[15]
miR1222	CGMMV	*C. lanatus*	Auxin-induced proteins, auxin-responsive proteins (CP)	Auxin signaling, translational activation of auxin-responsive genes	Induction of disease symptoms	[15]
miR1435	CGMMV	*C. lanatus*	Phenylpropanoid biosynthetic pathway genes (CP)	Phenylpropanoid synthesis (lignin, flavonoids, anthocyanins)	Plant growth and development, abiotic stress response, antimicrobial activity	[15]
miR2619	CGMMV	*C. lanatus*	ABC transporters (CP)	Intracellular transport	Mediating plant resistance to microbial attack	[15]
miR2655	CGMMV	*C. lanatus*	Auxin-induced proteins, auxin-responsive proteins (CP)	Auxin signaling, translational activation of auxin-responsive genes	Induction of disease symptoms	[15]
miR2662	CGMMV	*C. lanatus*	Sugar transporter (CP)	Intracellular transport	Enhances pathogen survival	[15]
miR2673	CGMMV	*C. sativus*	*Csa1M605660.1* (EE)	Growth of the pollen tube	Delayed flowering, reduced fruit production	[17]
miR2873	CGMMV	*C. lanatus*	Pectinesterase, pectinesterase inhibitors (CP)	Cell wall modulation, host–cell receptor recognition	Mediating plant resistance to microbial attack	[15]
miR2936	CGMMV	*C. sativus*	F-box protein encoding genes (EE)	Floral response to infection	Overexpression alters flowering time and seed formation in infected plants	[17,136]
miR3638	CGMMV	*C. sativus*	Unknown	Floral response to infection	Overexpression alters flowering time and seed formation in infected plants	[17]
miR4995	CGMMV	*C. lanatus*	*Cla010201*, *Cla017423* (CP)	Stress response	Response to CGMMV mediated by lncRNAs and circRNAs	[120]
miR5257	CGMMV	*C. lanatus*	Phenylpropanoid biosynthetic pathway genes (CP)	Phenylpropanoid synthesis (lignin, flavonoids, anthocyanins)	Plant growth and development, abiotic stress response, antimicrobial activity	[15]
miR5301	CMV	*S. lycopersicum*	TMV-resistance protein N-like gene, TIC 20-I protein gene	Recognition of specific pathogens and initiation of defense response through R genes	Plant immunity response	[123]
miR5303	CGMMV	*C. lanatus*	ABC transporters, cellulose synthase encoding genes, eukaryotic translation initiation factors, lipoxygenase A (CP)	Intracellular transport, cell wall modulation, cellulose synthesis, viral RNA replication, fatty acid signaling pathway	Mediating plant resistance to microbial attack, cell wall strengthening, response to virus infection	[15]
miR5368	CGMMV	*C. lanatus*	*Cla015442* (CP)	Stress response	Response to CGMMV mediated by lncRNAs and circRNAs	[120]
miR5637	CGMMV	*C. sativus*	Unknown	Unknown	Unknown	[137]
miR5658	CGMMV	*C. sativus*	LRR receptor-like serine/threonine-protein kinase FLS2-like (EE)	Mediates plant-pathogen interactions	Disease resistance	[16]
miR5741	CGMMV	*C. lanatus*	Ethylene-responsive factors, cytokinin dehydrogenase gene (CP)	Ethylene signaling, cytokinin signaling	Defense response	[15]
miR5827	CGMMV	*C. lanatus*	Phenylpropanoid biosynthetic pathway genes (CP)	Phenylpropanoid synthesis (lignin, flavonoids, anthocyanins)	Plant growth and development, abiotic stress response, antimicrobial activity	[15]
miR5998	CGMMV	*C. lanatus*	Sulfate transporter, sugar transporter, phenylpropanoid biosynthetic pathway genes (CP)	Intracellular transport, phenylpropanoid synthesis (lignin, flavonoids, anthocyanins)	Plant growth and development, abiotic stress response, antimicrobial activity, may enhance pathogen survival	[15]
miR6022	CMV	*S. lycopersicum*	*Hcr9-OR3A*	Recognition of specific pathogens and initiation of defense response through R genes	Plant immunity response	[123]
miR6114	CGMMV	*C. lanatus*	ABC transporters (CP)	Intracellular transport	Mediating plant resistance to microbial attack	[15]
miR6300	CGMMV	*C. lanatus*	*Cla009402*, *Cla010518*, *Cla014433*, *Cla019496*, *Cla020836* (CP)	Stress response	Response to CGMMV mediated by lncRNAs and circRNAs	[120]
miR6455	CGMMV	*C. lanatus*	ABC transporters (CP)	Intracellular transport	Mediating plant resistance to microbial attack	[15]
miR7517	CGMMV	*C. lanatus*	Sucrose transporters (CP)	Intracellular transport	Natural resistance to viruses, inhibits viral replication and movement	[15]
miR8051	CGMMV	*C. lanatus*	Long-chain-fatty-acid-CoA ligase (CP)	Fatty acid signaling pathway	Response to virus infection	[15]
miR8578.1	CGMMV	*C. lanatus*	Pectinesterase, pectinesterase inhibitors, auxin-induced proteins, auxin-responsive proteins, lipoxygenase A (CP)	Cell wall modulation, host–cell receptor recognition, auxin signaling, translational activation of auxin-responsive genes, fatty acid signaling pathway	Mediating plant resistance to microbial attack, induction of disease symptoms, response to virus infection	[15]
miR9410	CGMMV	*C. lanatus*	*Cla012690*, *Cla013592* (CP)	Stress response	Response to CGMMV mediated by lncRNAs and circRNAs	[120]
miR9752	CGMMV	*C. lanatus*	Auxin-induced proteins, auxin-responsive proteins (CP)	Auxin signaling, translational activation of auxin-responsive genes	Induction of disease symptoms	[15]
miR11610	CGMMV	*C. lanatus*	*Cla023097* (CP)	Stress response	Response to CGMMV mediated by lncRNAs and circRNAs	[120]
csa-miRn1-3p	CGMMV	*C. sativus*	*MYB* genes, UDP-glycosyltransferase 73B3-like (EE)	Cellular morphogenesis and development, secondary metabolism, biotic and abiotic stress response, stress signaling, meristem formation, cell cycle, induction of UDP-glycosyltransferase and scopoletin glucosyltransferase	Pathogen resistance	[16,17]
csa-miRn2-3p	CGMMV	*C. sativus*	*bZIP* genes (EE)	Light and stress signaling, flower development	Pathogen resistance, involved in smaller regulatory subnetworks	[16,17]
csa-miRn3-3p	CGMMV	*C. sativus*	*Csa4M578870.1* (EE)	Cell wall metabolism, growth, differentiation	Plant dwarfism, involved in smaller regulatory subnetworks	[16,17]
csa-miRn4-5p	CGMMV	*C. sativus*	Unknown	Unknown	Involved in smaller regulatory subnetworks	[16,17]
csa-miRn5-5p	CGMMV	*C. sativus*	pectin acetylesterase, glucan endo-1,3-beta-glucosidase, basic isoform-like, *Csa2M033380.1* (EE)	Cell wall metabolism, actin-binding, regulation of cytoskeletal microfilaments, involved in fungal pathogen defense	Cell wall degradation, plant defense	[16,17]
csa-miRn6-3p	CGMMV	*C. sativus*	Ethylene-responsive transcription factor *CRF4*-like, Putative disease resistance protein RGA4- like, pathogenesis-related homeodomain protein-like, vicilin-like antimicrobial peptides 2-1-like, linoleate 9S-lipoxygenase 1-like, RNA polymerase II C-terminal domain phosphatase-like 3-like, probable dehydrin LEA-like, *Csa2M033380.1*, *Csa7M219220.1*, *Csa3M683670.1*, *Csa3M117970.1*, *Csa3M019980.1*, *Csa2M380020.2*, *Csa3M119700.1*, *Csa4M000700.1* (EE)	Lipid biosynthetic/metabolic processes, acyltransferase activity, zinc ion binding, encoding constituents of the thylakoid membrane, plant growth, development and senescence, cold and water stress response, phytohormone response	Mottling of leaves, defense response, disease resistance, pathogen and pest resistance, regulation of growth and stress response, regulation of genes by stress factors and signal transduction pathways	[16,17]
csa-miRn7-5p	CGMMV	*C. sativus*	Unknown	Unknown	Unknown	[16,17]
csa-miRn8-3p	CGMMV	*C. sativus*	*Csa7M257340.1* (EE)	Nitrate transporter	Affects crop yield, involved in smaller regulatory subnetworks	[16,17]
novel_54	CGMMV	*C. lanatus*	*Cla019485* (CP)	Stress response	Response to CGMMV mediated by circRNAs	[120]

Species: *C. sativus*—*Cucumis sativus* (cucumber), *C. lanatus*—*Citrullus lanatus* (watermelon), *A. thaliana*—*Arabidopsis thaliana*, *C. annum*—*Capsicum annum* (hot pepper), *N. benthamiana*—*Nicotiana benthamiana*, *N. tabacum*—*Nicotiana tabacum* (tobacco), *S. lycopersicum*—*Solanum lycopersicum* (tomato).

## Data Availability

No new data was created or analyzed in this study. Data sharing is not applicable to this article.
